# Supplementary material for: Radical transformation pathway towards sustainable electricity via evolutionary steps
Source: Nat Commun. 2019 Mar 6;10:1077. doi: 10.1038/s41467-019-08855-1 (PMC6403340; doi:10.1038/s41467-019-08855-1)
Supplement: Supplementary file 1 — Supplementary Information [file 41467_2019_8855_MOESM1_ESM.pdf]

Supplementary Information for

Pathway towards sustainable electricity - Radical transformation via evolutionary steps

Dmitrii Bogdanov et al.
Correspondence to: Dmitrii.Bogdanov@lut.fi and Christian.Breyer@lut.fi.

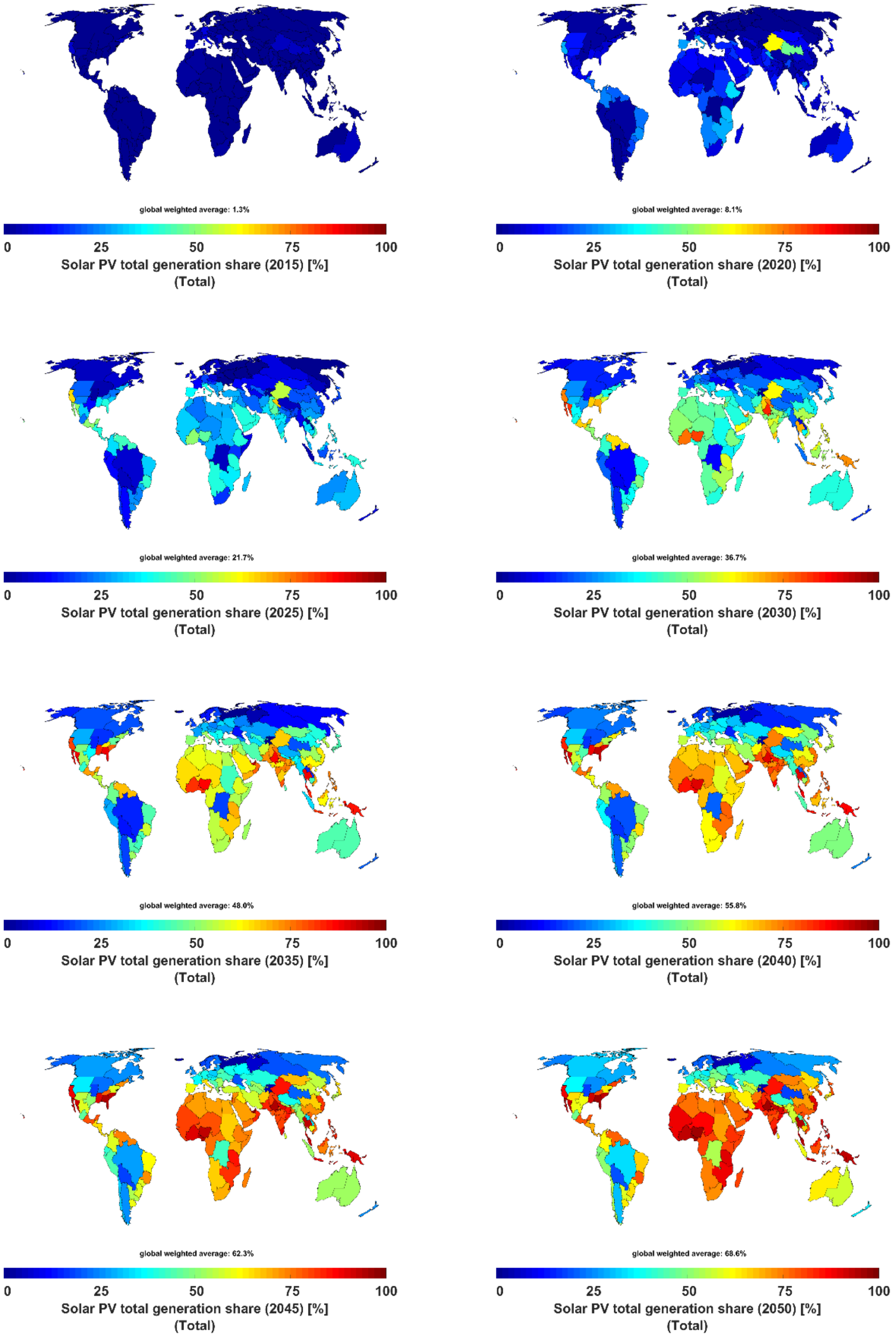

Supplementary Figure 1. PV generation shares for all transition steps.

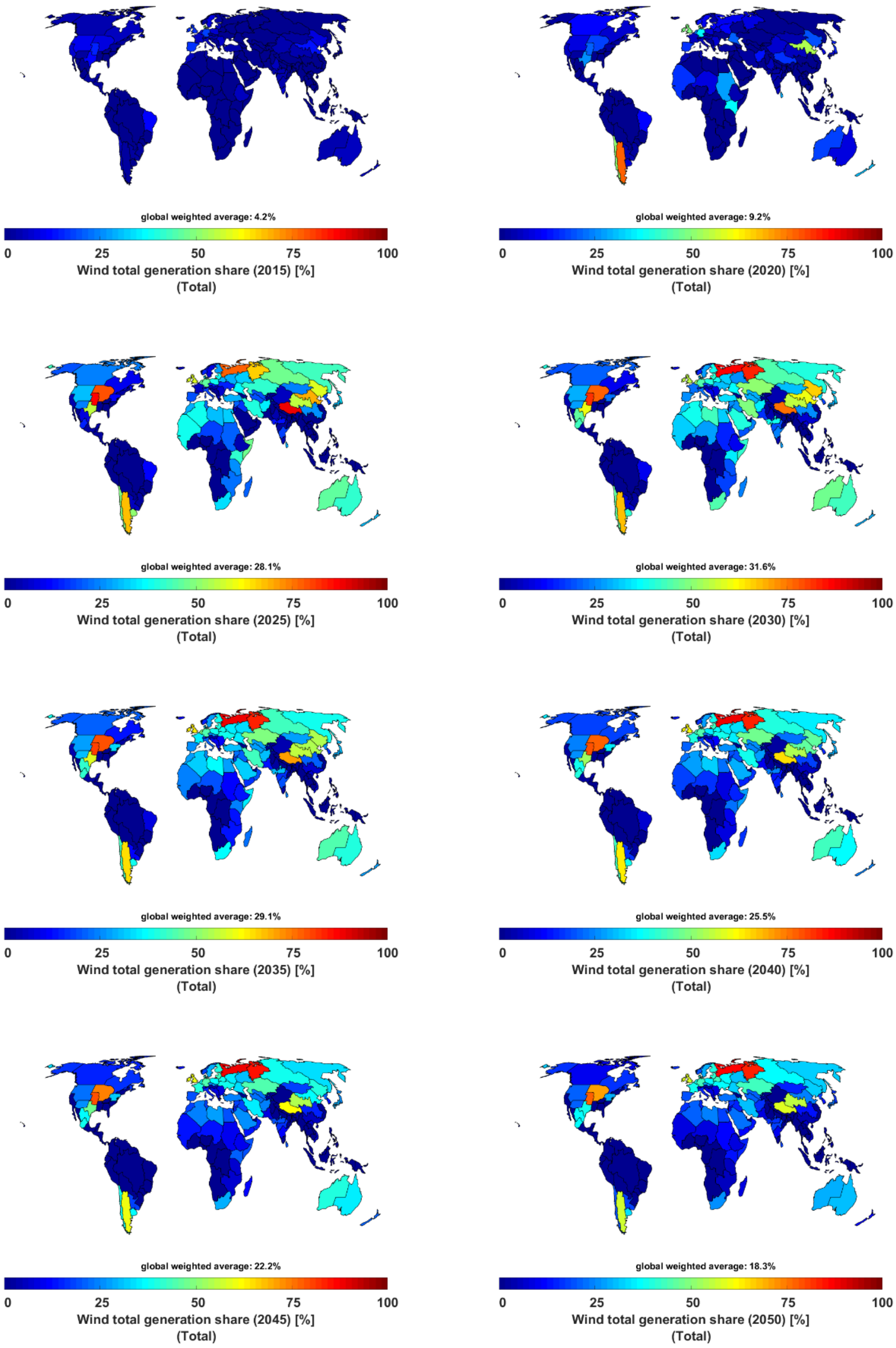

Supplementary Figure 2. Wind generation shares for all transition steps.

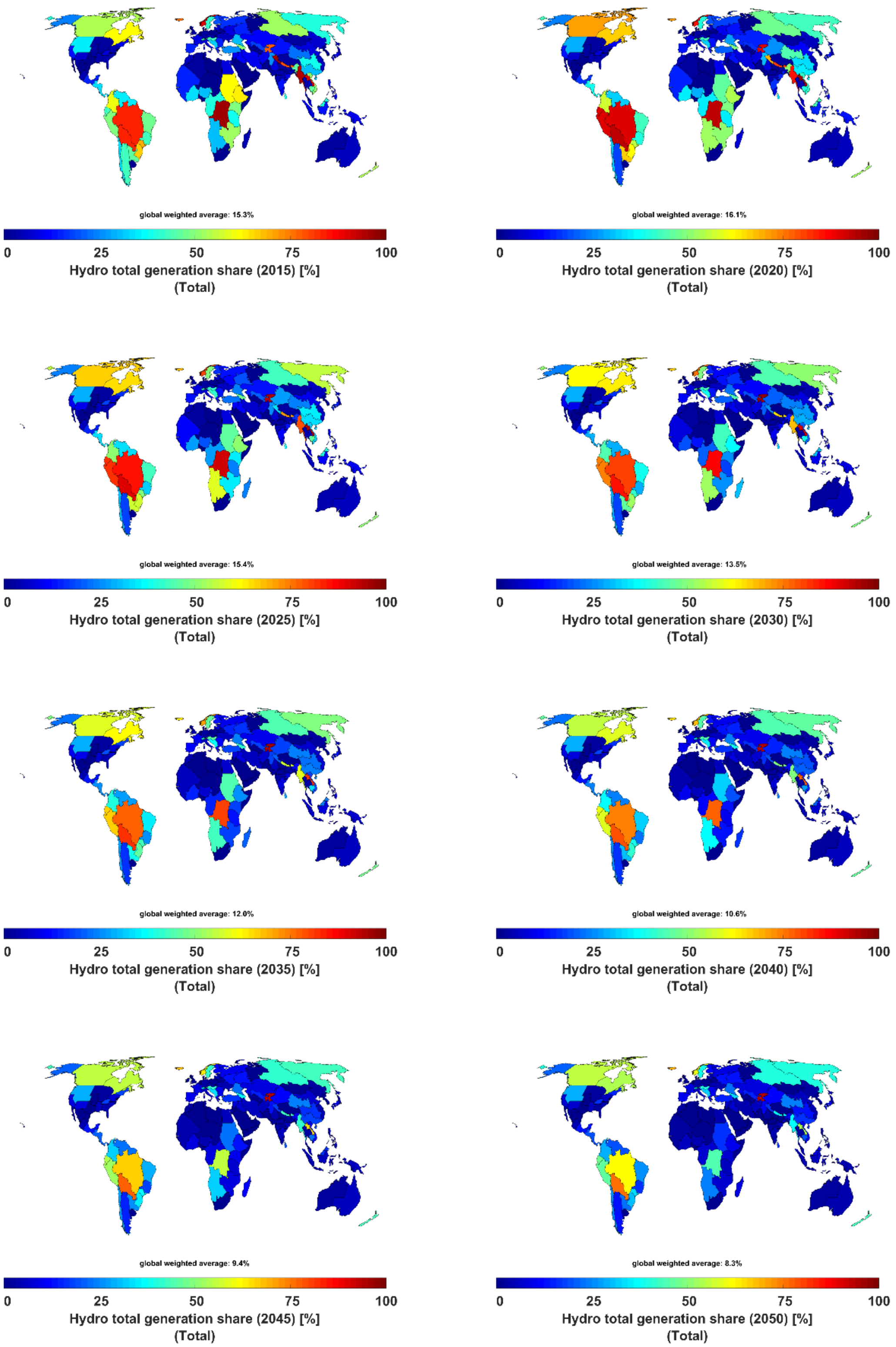

Supplementary Figure 3. Hydro generation shares for all transition steps.

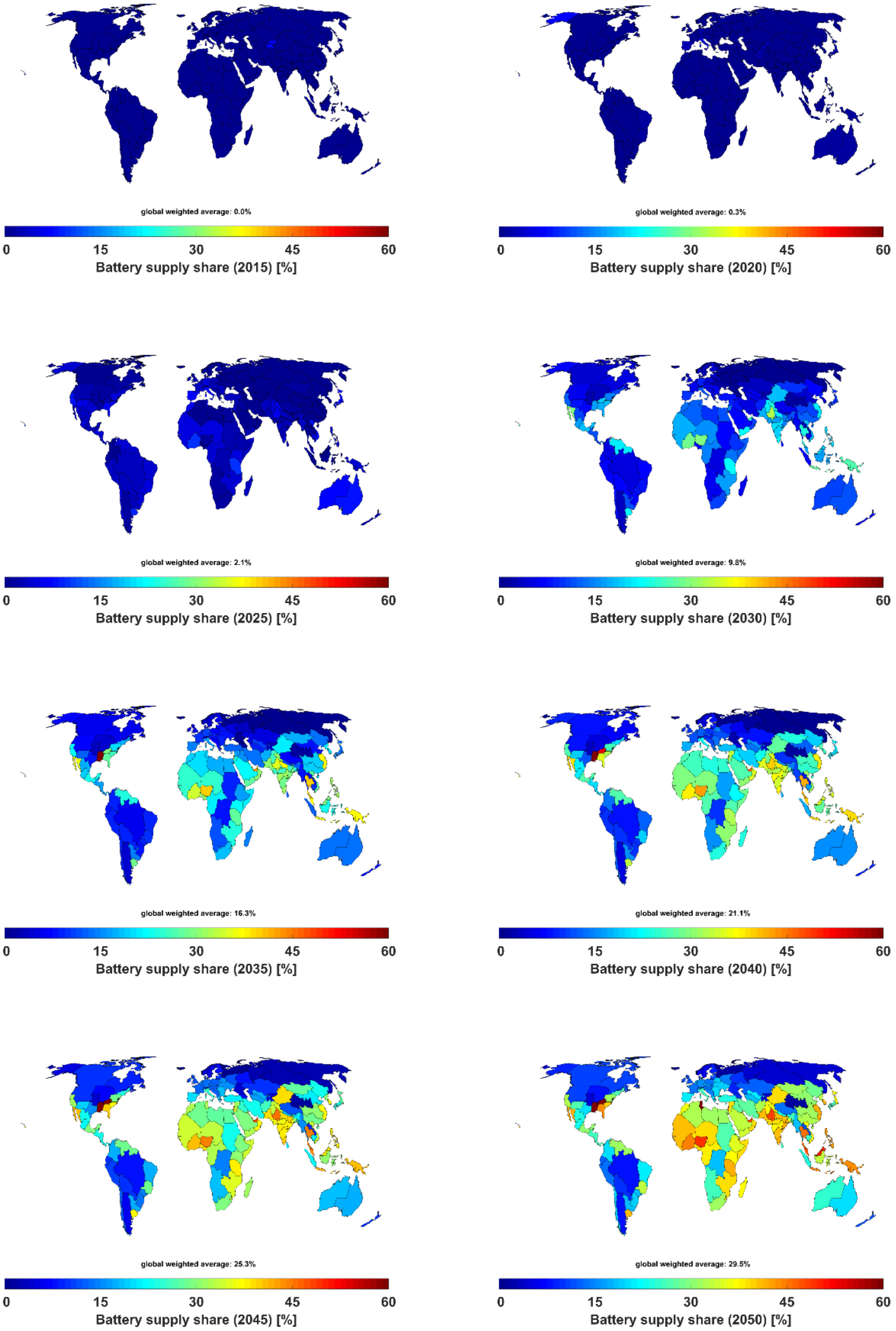

Supplementary Figure 4. Batteries supply shares for all transition steps.

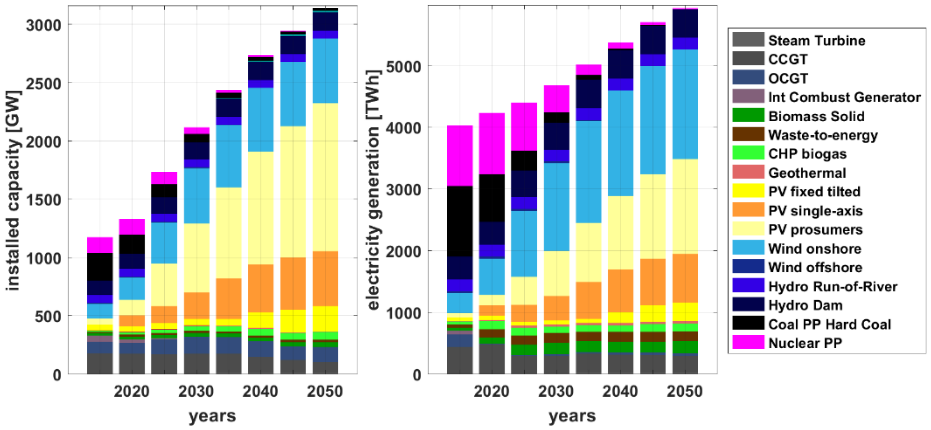

Supplementary Figure 5. Europe - Installed capacities and generation by technologies during the energy transition from 2015 to 2050.

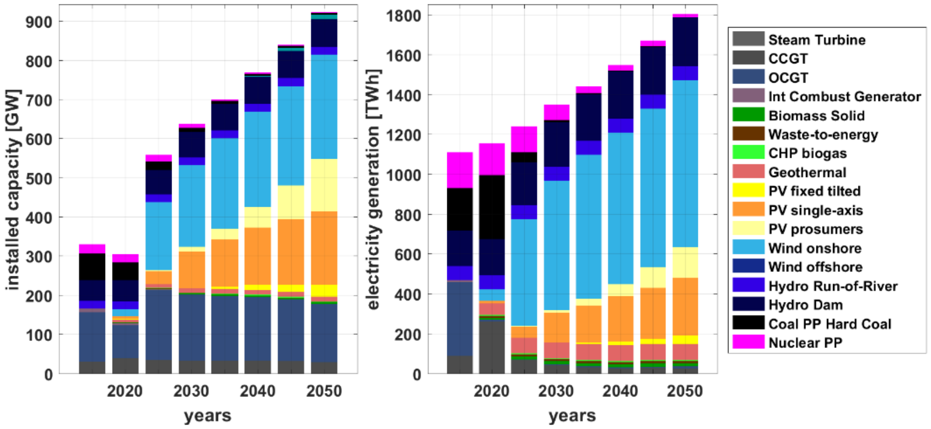

Supplementary Figure 6. Eurasia - Installed capacities and generation of power and storage technologies during the energy transition from 2015 to 2050.

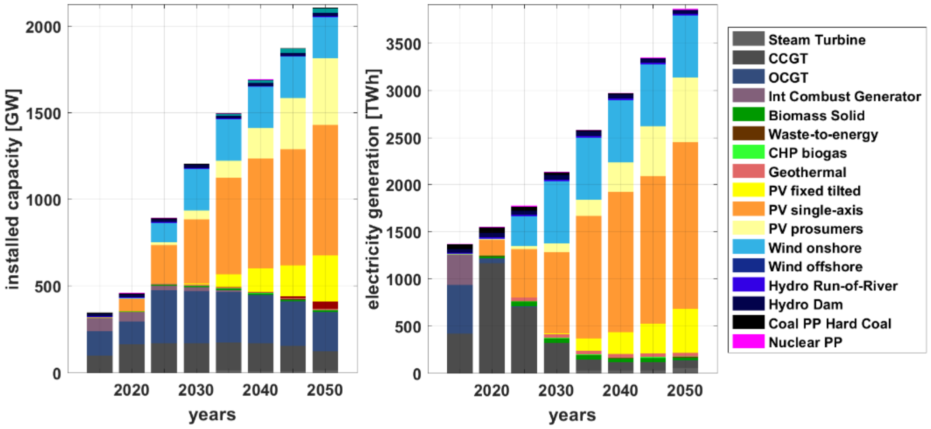

Supplementary Figure 7. MENA - Installed capacities and generation of power and storage technologies during the energy transition from 2015 to 2050.

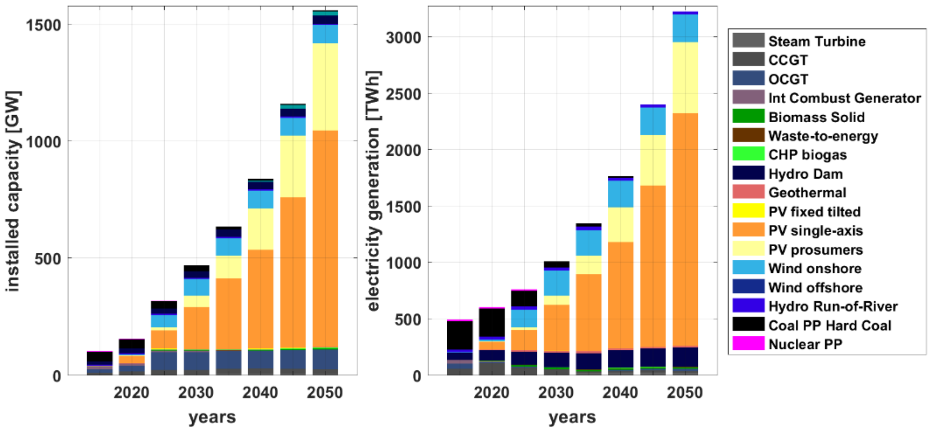

Supplementary Figure 8. Sub-Saharan Africa - Installed capacities and generation of power and storage technologies during the energy transition from 2015 to 2050.

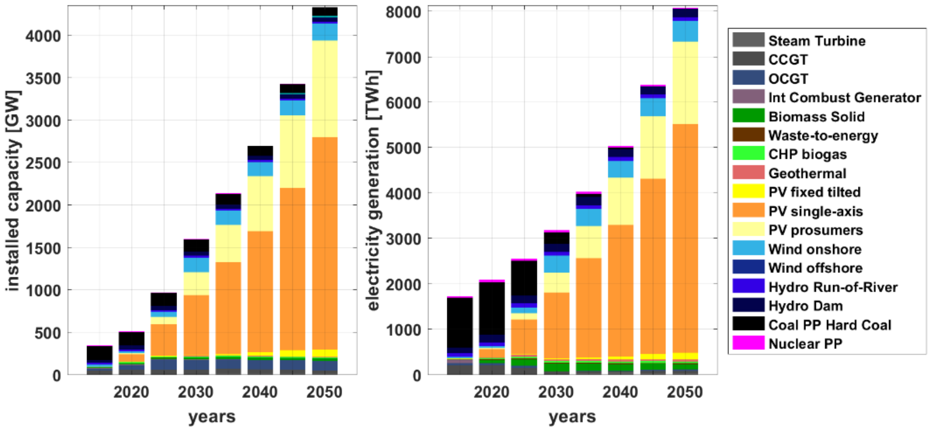

Supplementary Figure 9. SAARC - Installed capacities and generation of power and storage technologies during the energy transition from 2015 to 2050.

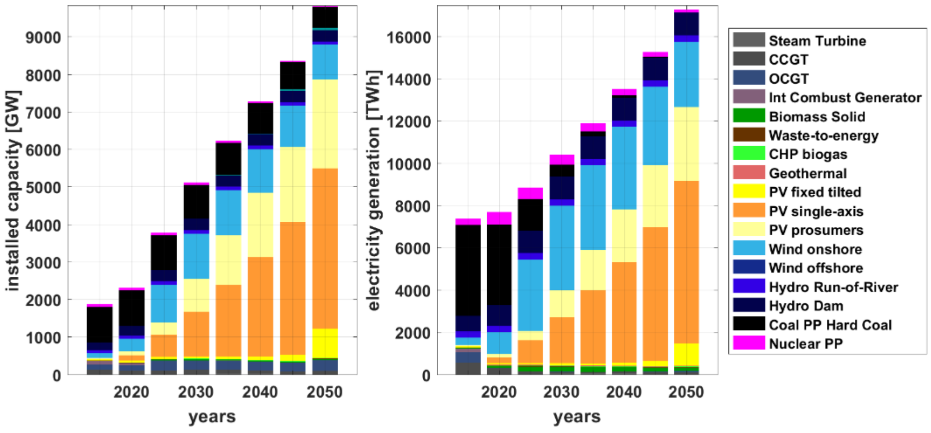

Supplementary Figure 10. Northeast Asia - Installed capacities and generation of power and storage technologies during the energy transition from 2015 to 2050.

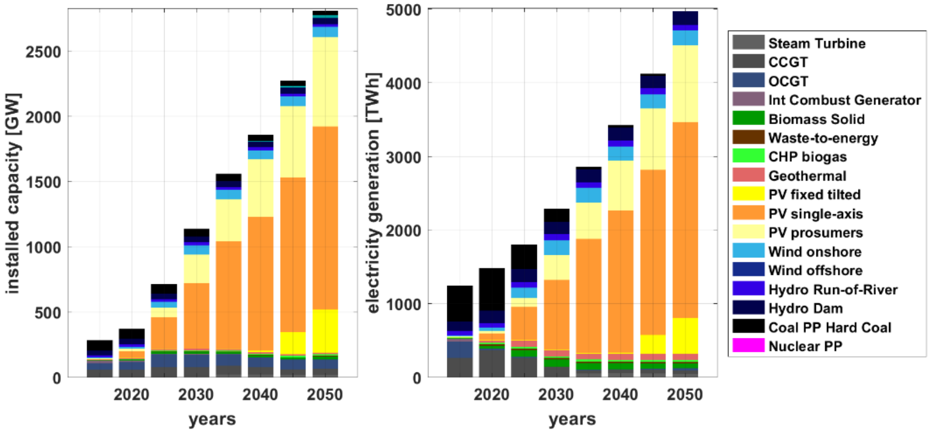

Supplementary Figure 11. Southeast Asia - Installed capacities and generation of power and storage technologies during the energy transition from 2015 to 2050.

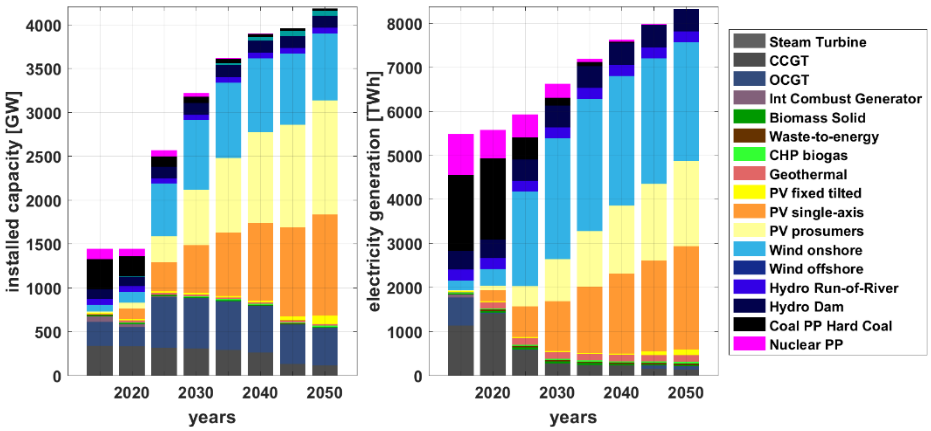

Supplementary Figure 12. North America - Installed capacities and generation of power and storage technologies during the energy transition from 2015 to 2050.

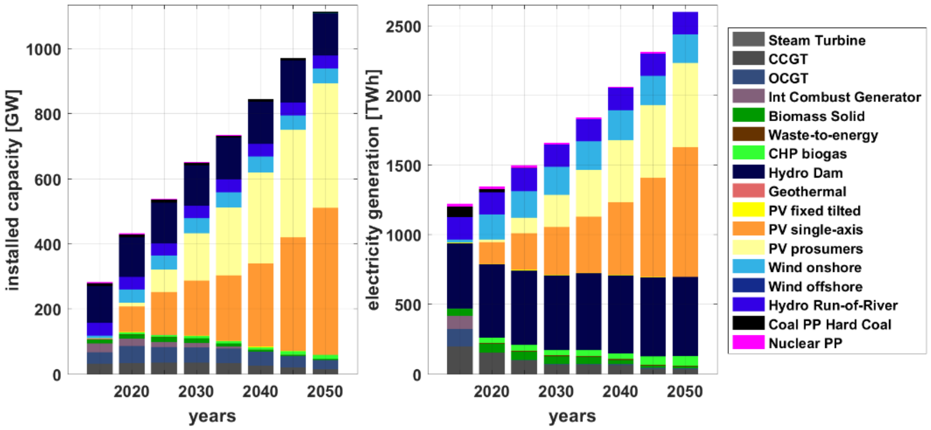

Supplementary Figure 13. South America - Installed capacities and generation of power and storage technologies during the energy transition from 2015 to 2050.

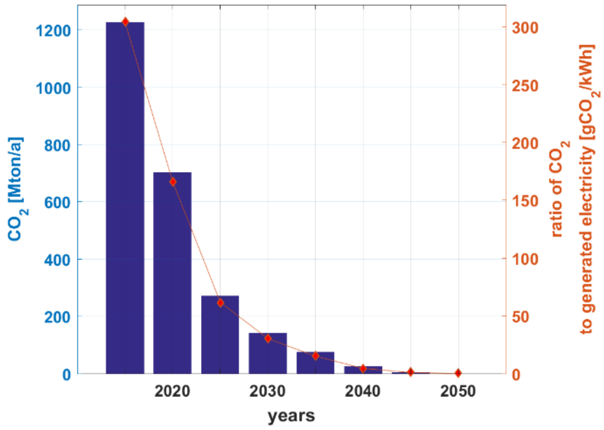

Supplementary Figure 14. Europe - CO_2 emissions during the energy transition from 2015 to 2050.

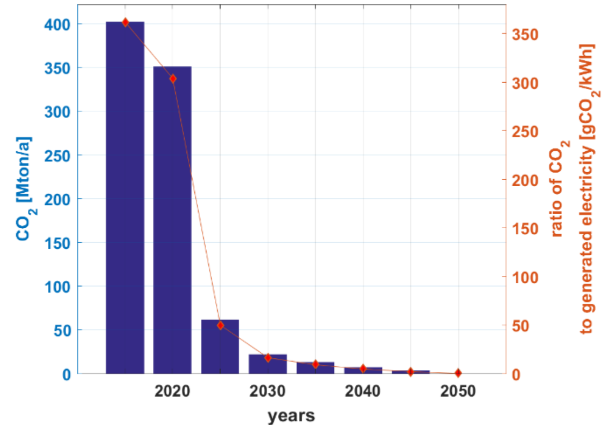

Supplementary Figure 15. Eurasia - CO_2 emissions during the energy transition from 2015 to 2050.

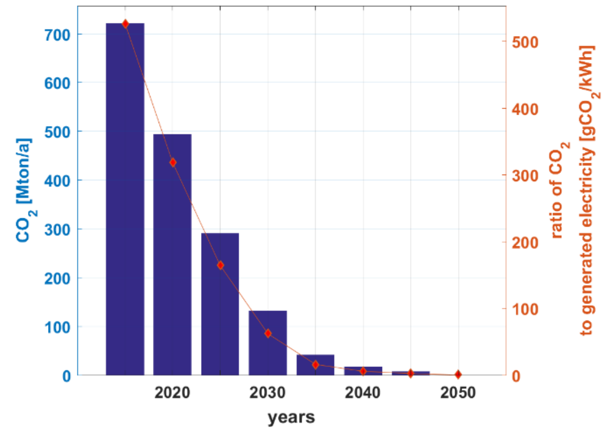

Supplementary Figure 16. MENA - CO_2 emissions during the energy transition from 2015 to 2050.

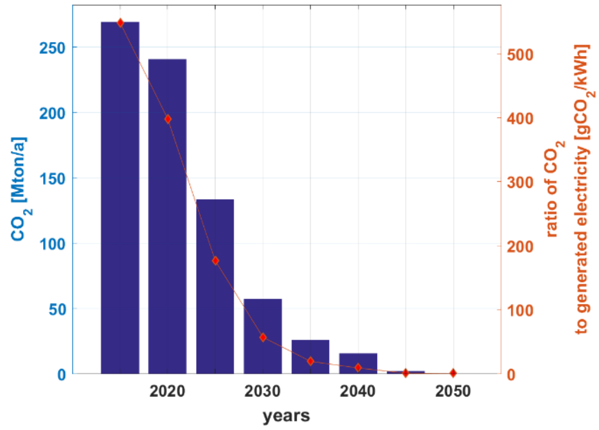

Supplementary Figure 17. Sub-Saharan Africa - CO_2 emissions during the energy transition from 2015 to 2050.

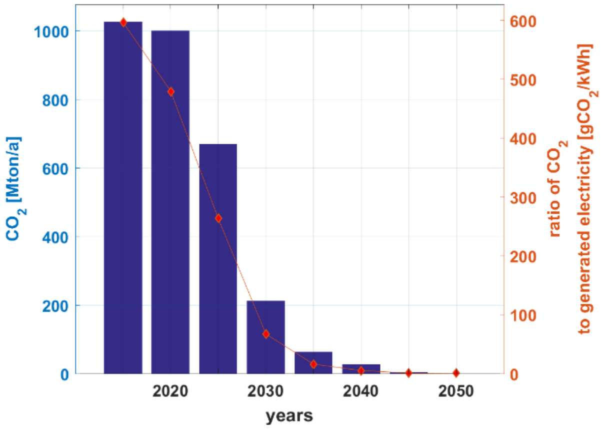

Supplementary Figure 18. SAARC - CO_2 emissions during the energy transition from 2015 to 2050.

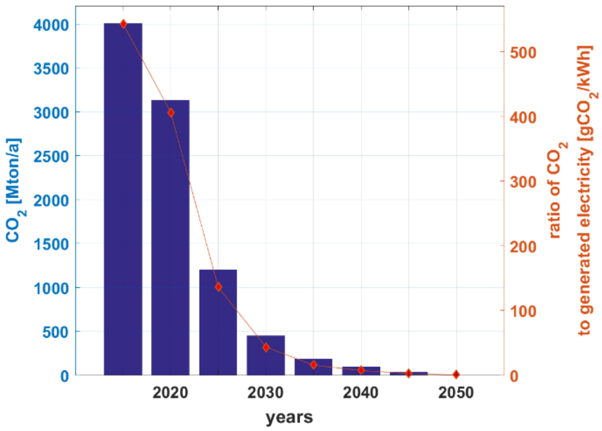

Supplementary Figure 19. Northeast Asia - CO_2 emissions during the energy transition from 2015 to 2050.

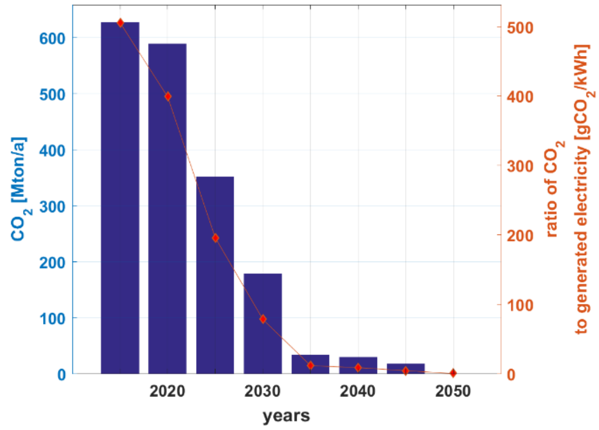

Supplementary Figure 20. Southeast Asia - CO_2 emissions during the energy transition from 2015 to 2050.

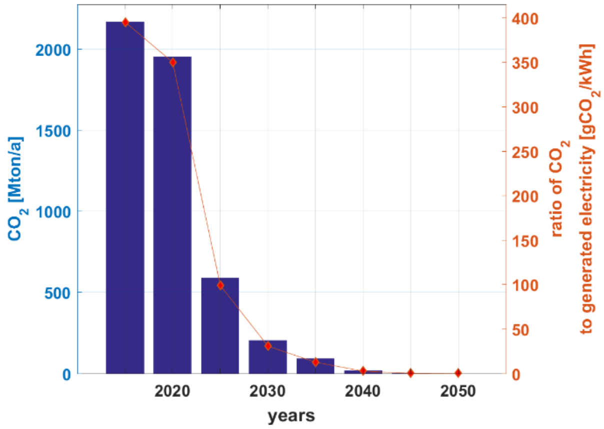

Supplementary Figure 21. North America - CO_2 emissions during the energy transition from 2015 to 2050.

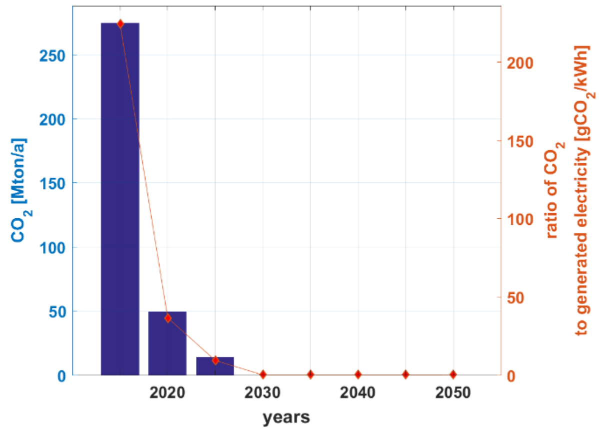

Supplementary Figure 22. South America - CO_2 emissions during the energy transition from 2015 to 2050.

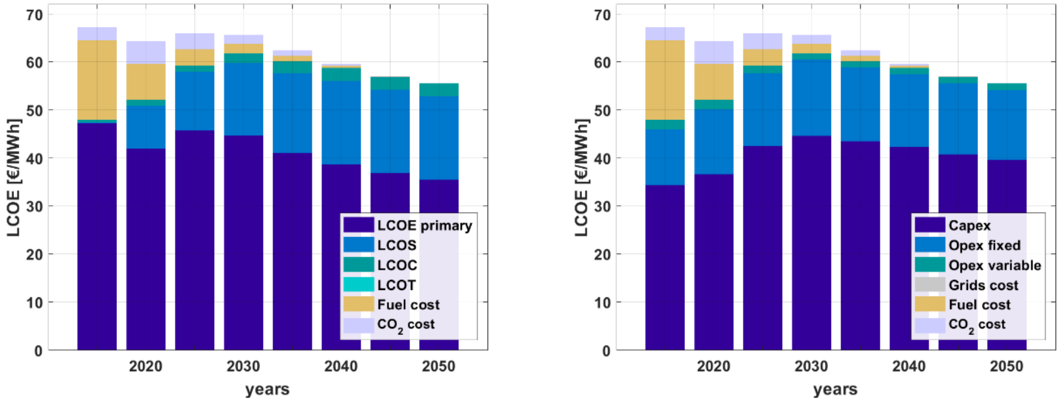

Supplementary Figure 23. Europe - LCOE breakdown during the energy transition from 2015 to 2050.

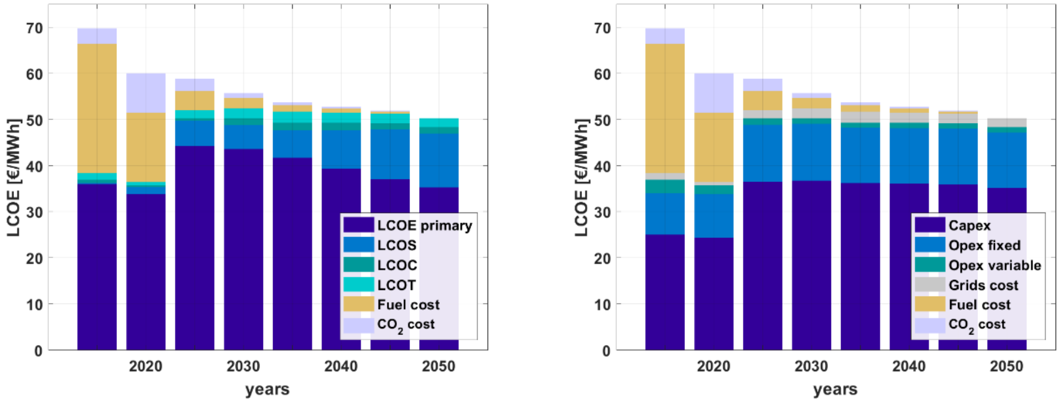

Supplementary Figure 24. Eurasia - LCOE breakdown during the energy transition from 2015 to 2050.

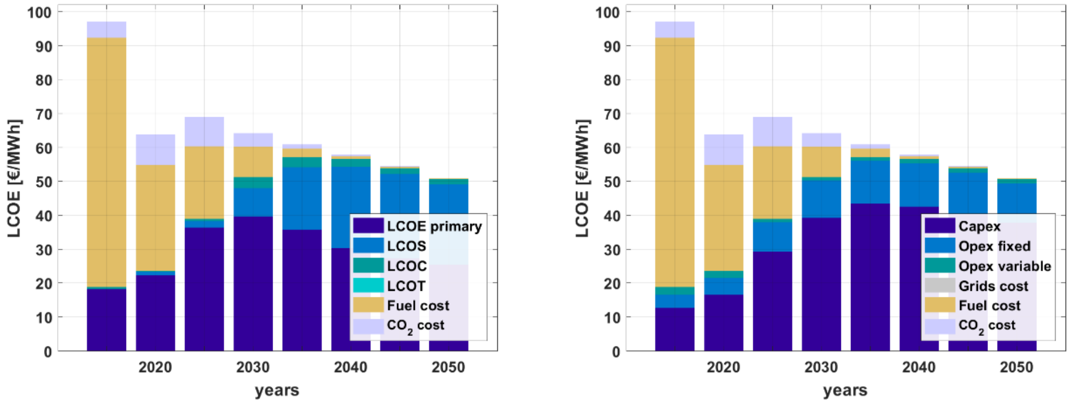

Supplementary Figure 25. MENA - LCOE breakdown during the energy transition from 2015 to 2050.

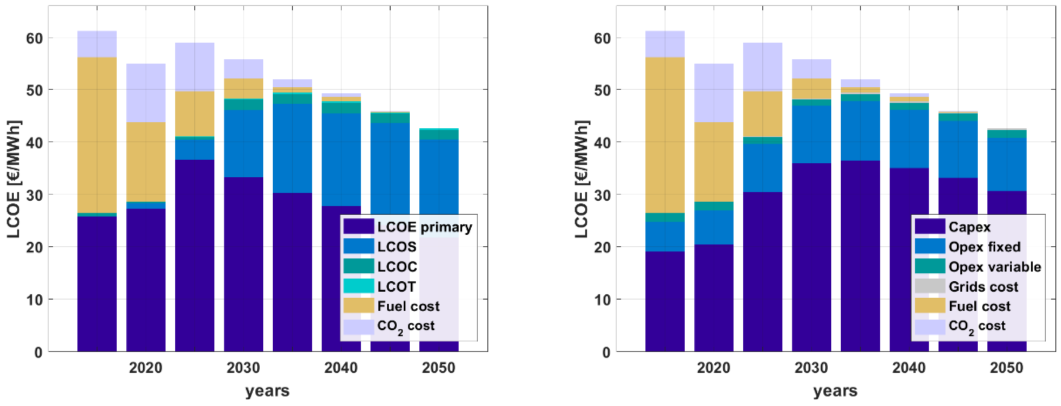

Supplementary Figure 26. Sub-Saharan Africa - LCOE breakdown during the energy transition from 2015 to 2050.

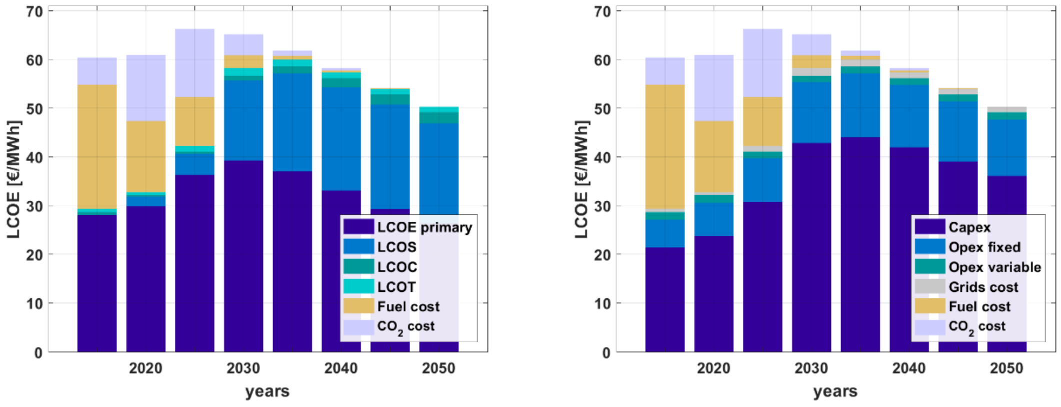

Supplementary Figure 27. SAARC - LCOE breakdown during the energy transition from 2015 to 2050.

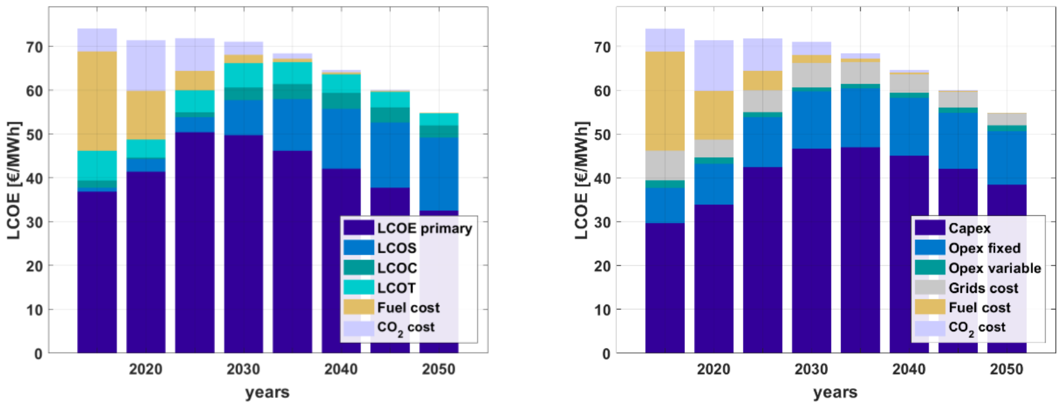

Supplementary Figure 28. Northeast Asia - LCOE breakdown during the energy transition from 2015 to 2050.

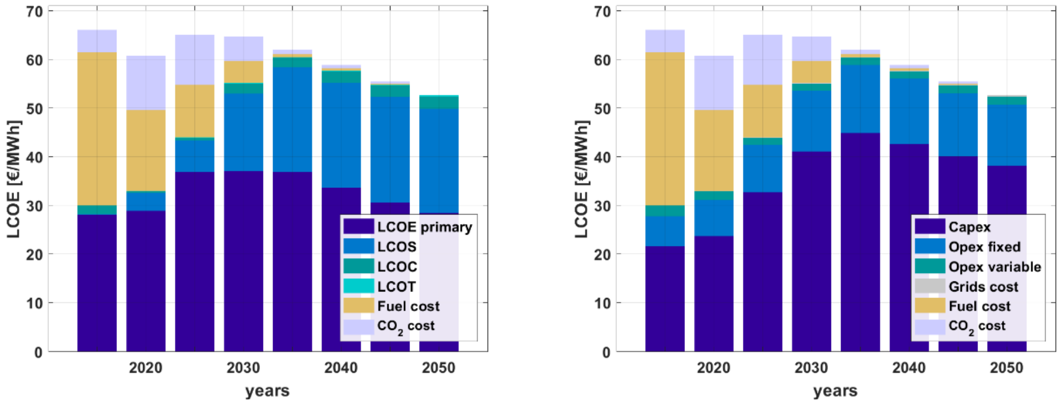

Supplementary Figure 29. Southeast Asia - LCOE breakdown during the energy transition from 2015 to 2050.

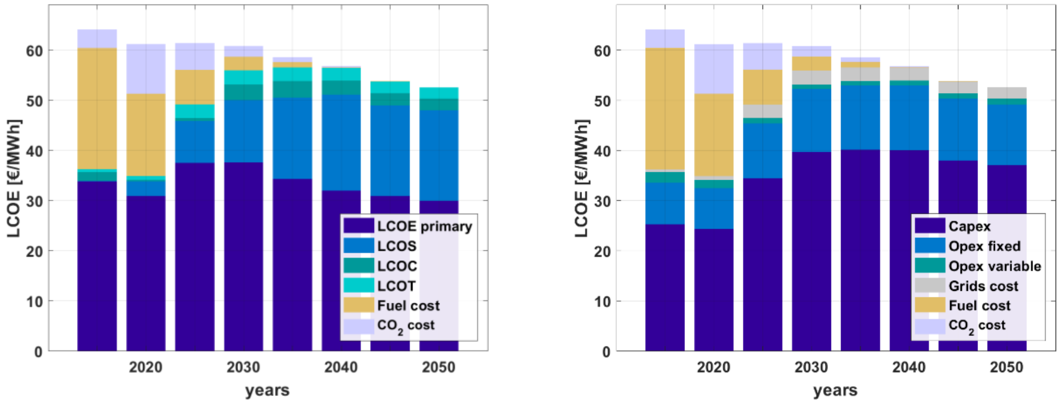

Supplementary Figure 30. North America - LCOE breakdown during the energy transition from 2015 to 2050.

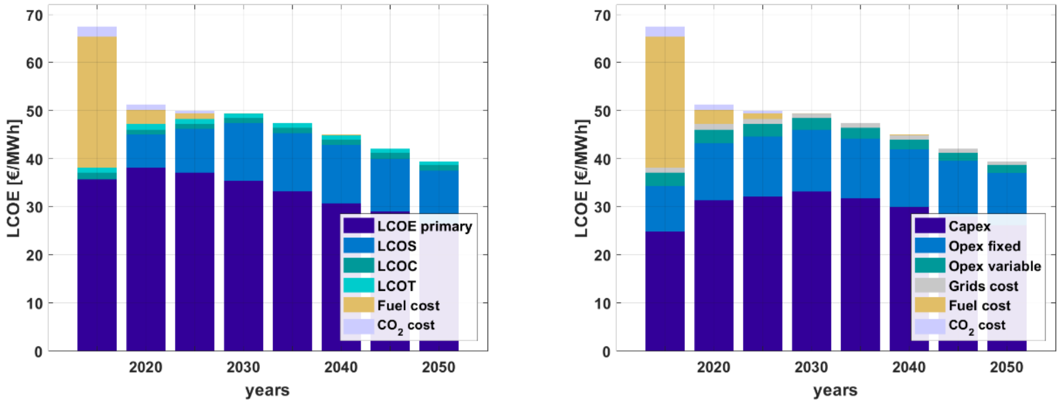

Supplementary Figure 31. South America - LCOE breakdown during the energy transition from 2015 to 2050.

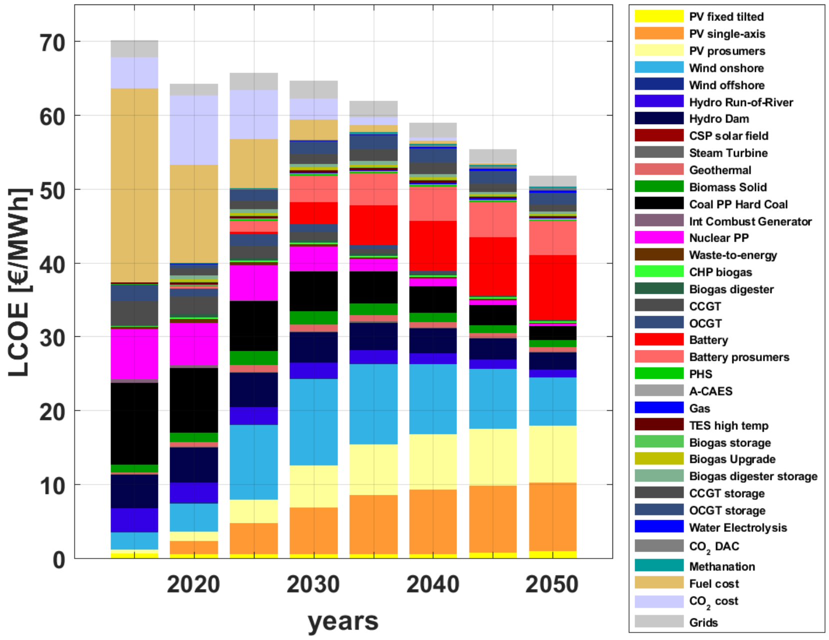

Supplementary Figure 32. Global - LCOE breakdown by technology during the energy transition from 2015 to 2050.

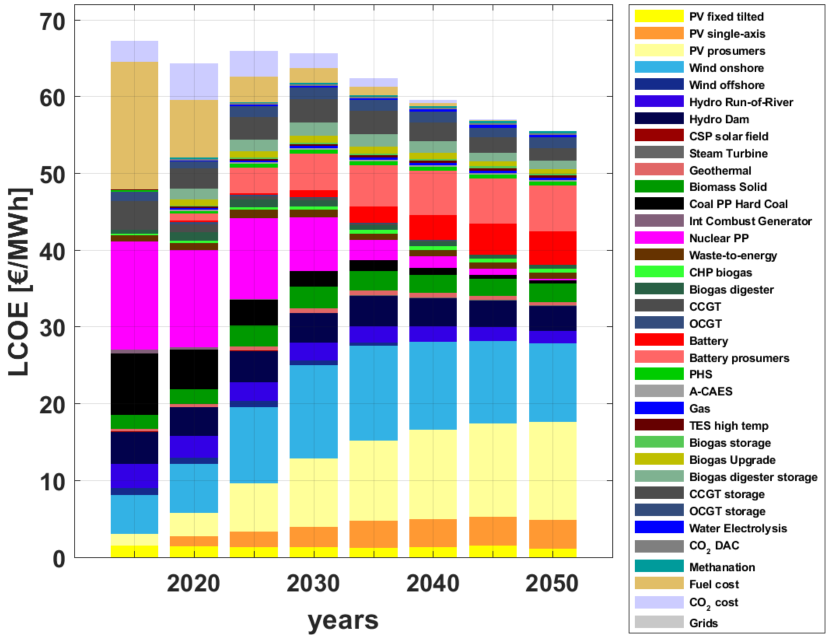

Supplementary Figure 33. Europe - LCOE breakdown by technology during the energy transition from 2015 to 2050.

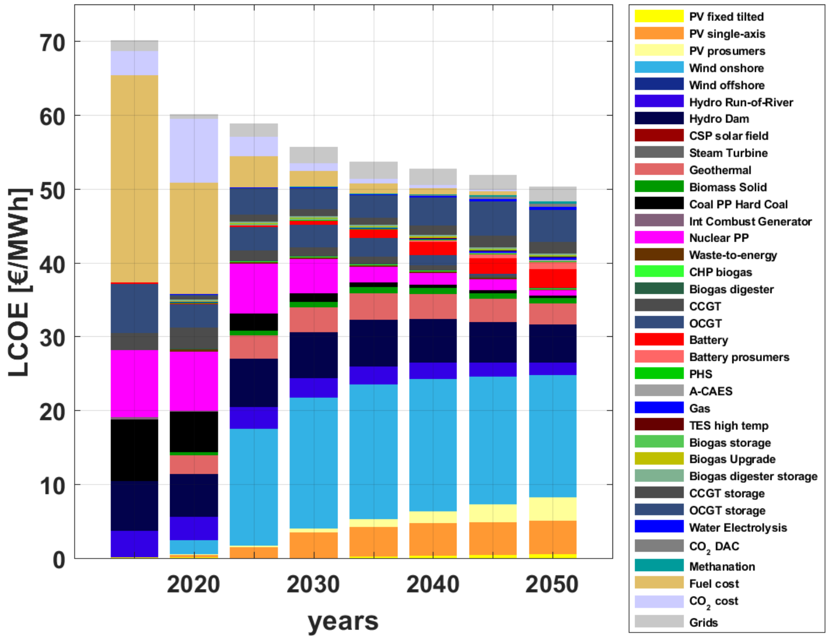

Supplementary Figure 34. Eurasia - LCOE breakdown by technology during the energy transition from 2015 to 2050.

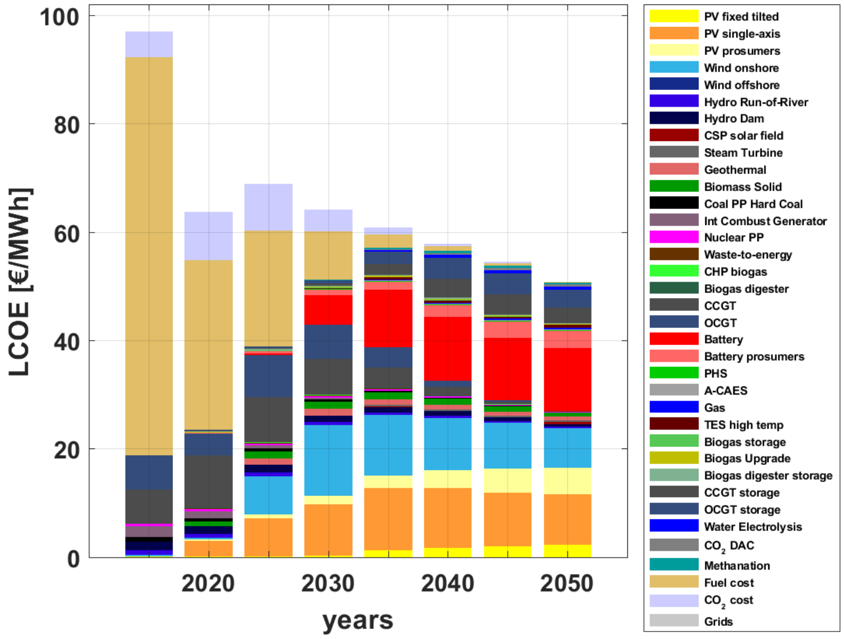

Supplementary Figure 35. MENA - LCOE breakdown by technology during the energy transition from 2015 to 2050.

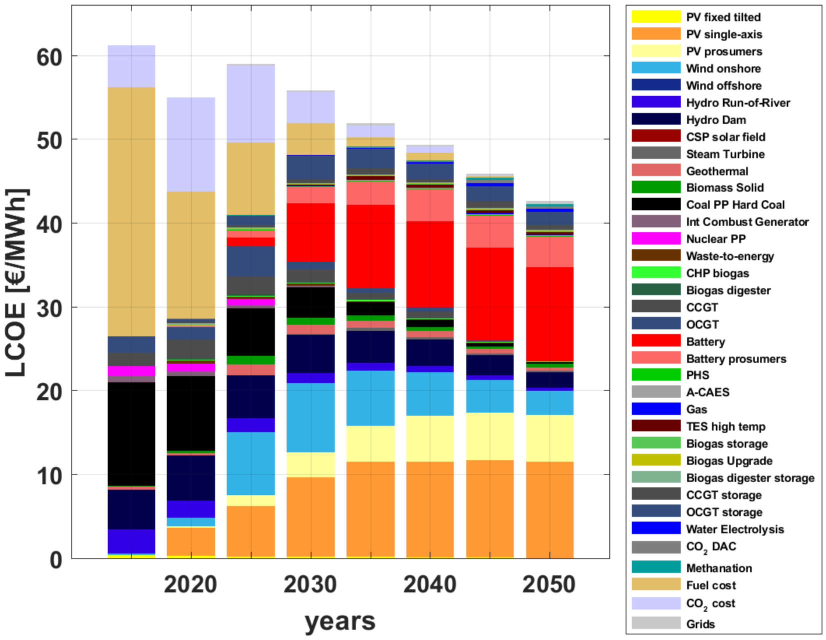

Supplementary Figure 36. Sub-Saharan Africa - LCOE breakdown by technology during the energy transition from 2015 to 2050.

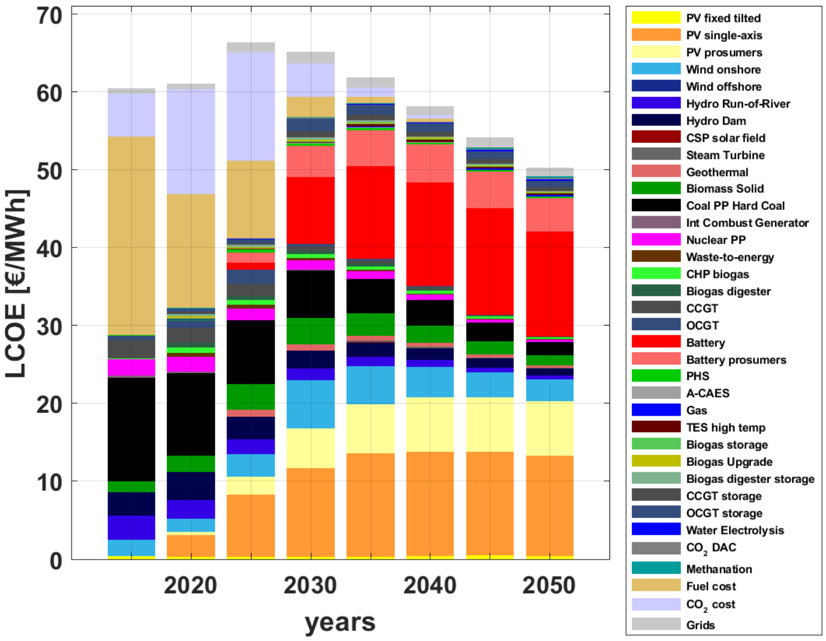

Supplementary Figure 37. SAARC - LCOE breakdown by technology during the energy transition from 2015 to 2050.

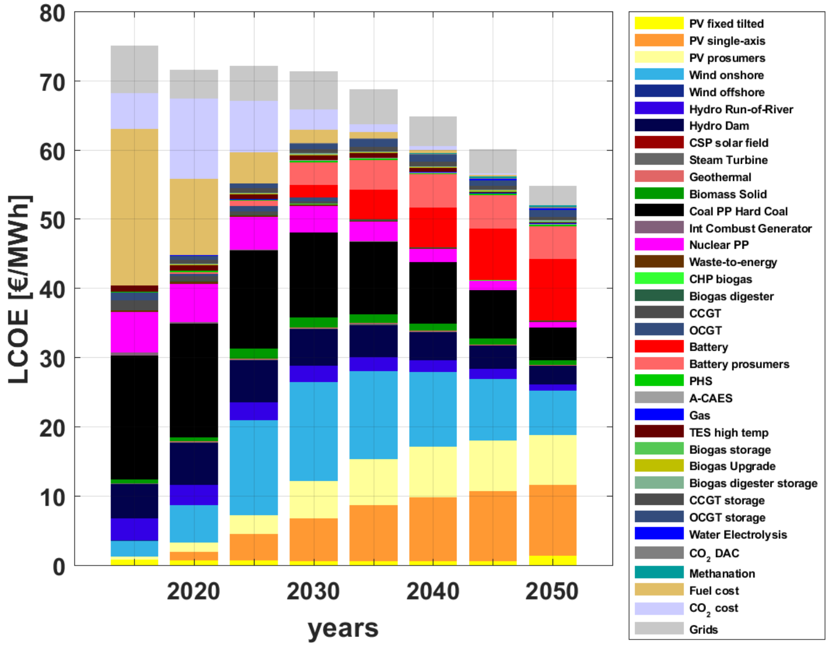

Supplementary Figure 38. Northeast Asia - LCOE breakdown by technology during the energy transition from 2015 to 2050.

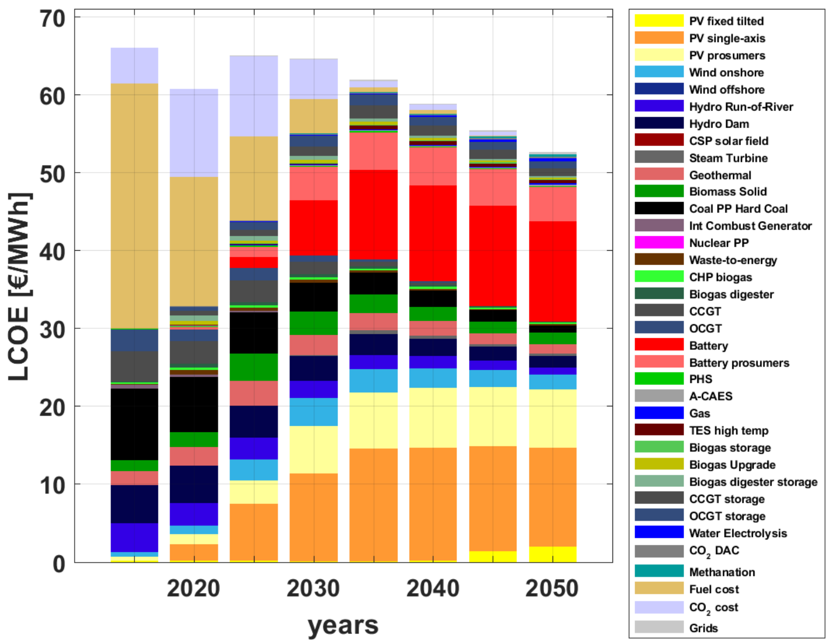

Supplementary Figure 39. Southeast Asia - LCOE breakdown by technology during the energy transition from 2015 to 2050.

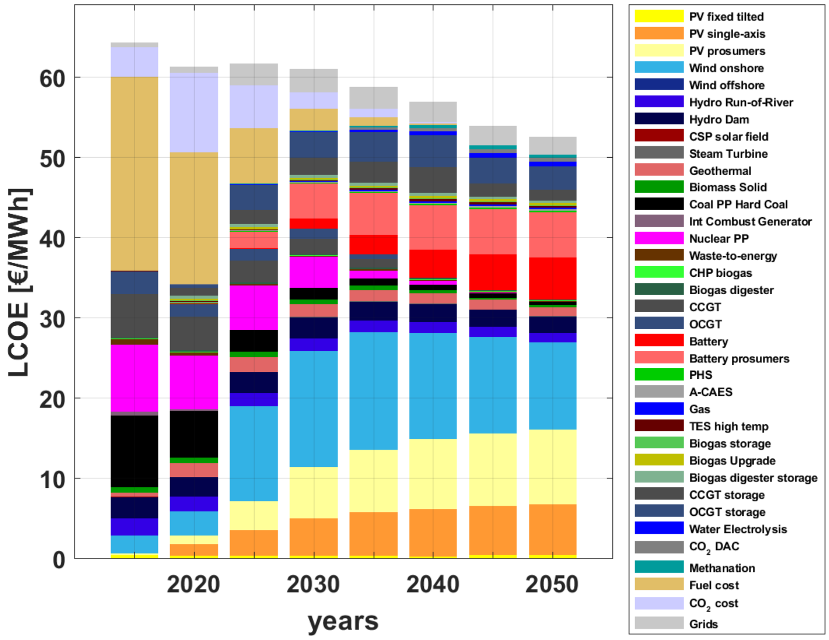

Supplementary Figure 40. North America - LCOE breakdown by technology during the energy transition from 2015 to 2050.

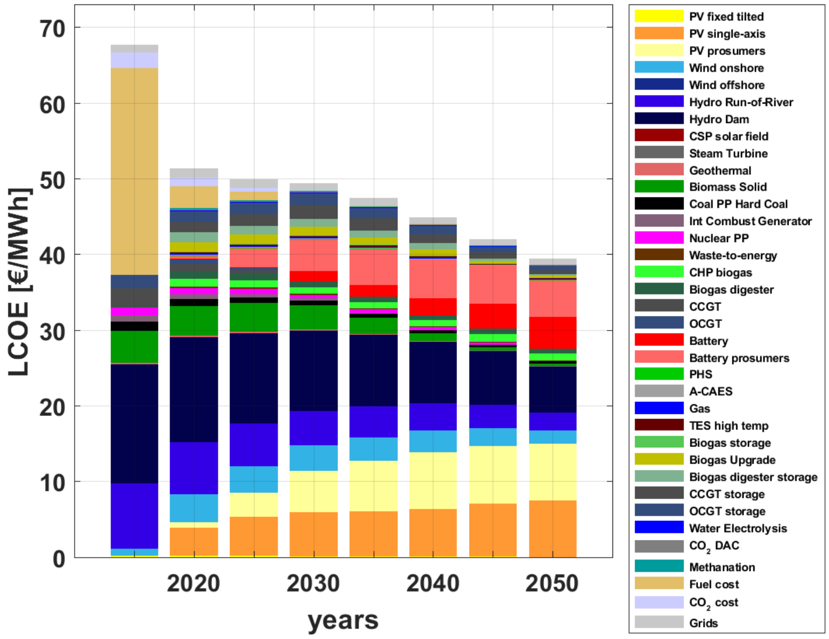

Supplementary Figure 41. South America - LCOE breakdown by technology during the energy transition from 2015 to 2050.

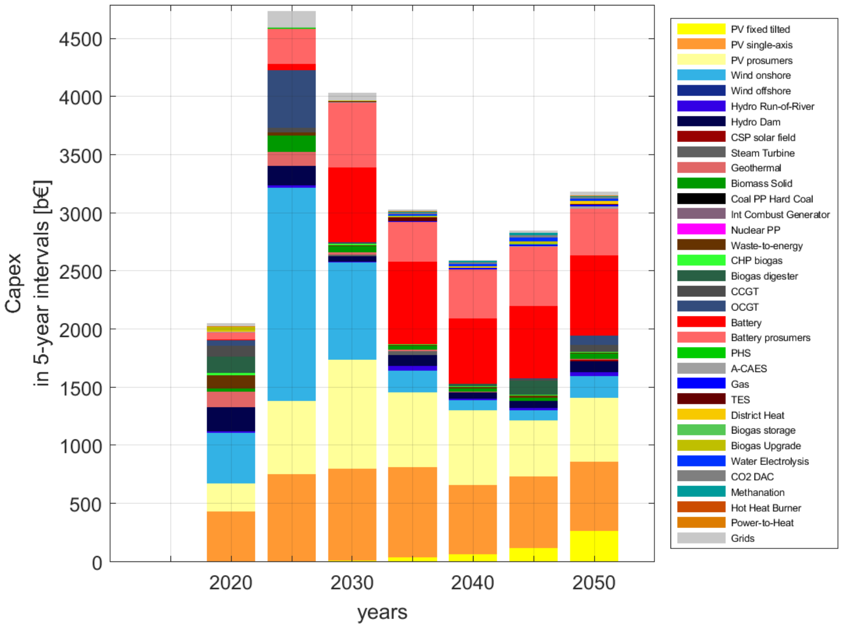

Supplementary Figure 42. Global - CAPEX breakdown by technology during the energy transition from 2015 to 2050.

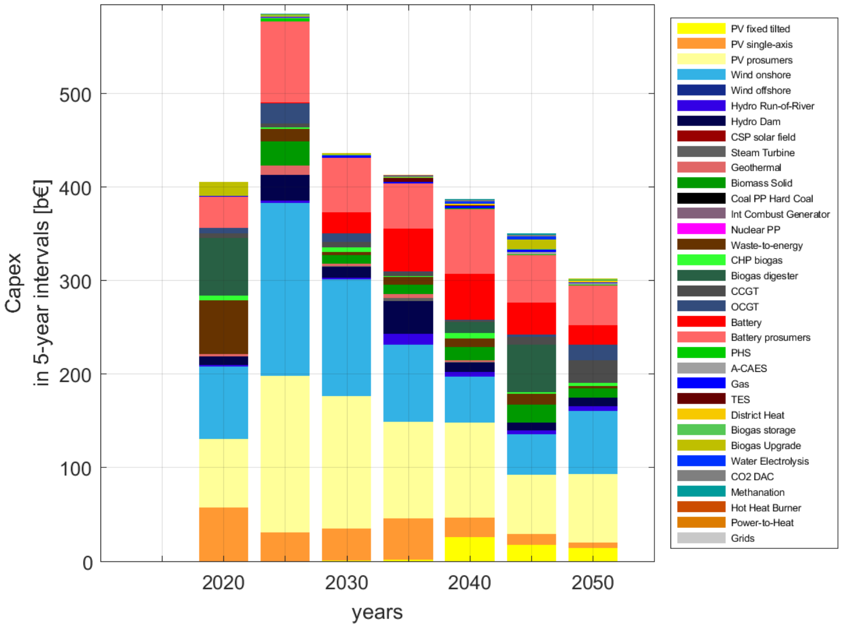

Supplementary Figure 43. Europe - CAPEX breakdown by technology during the energy transition from 2015 to 2050.

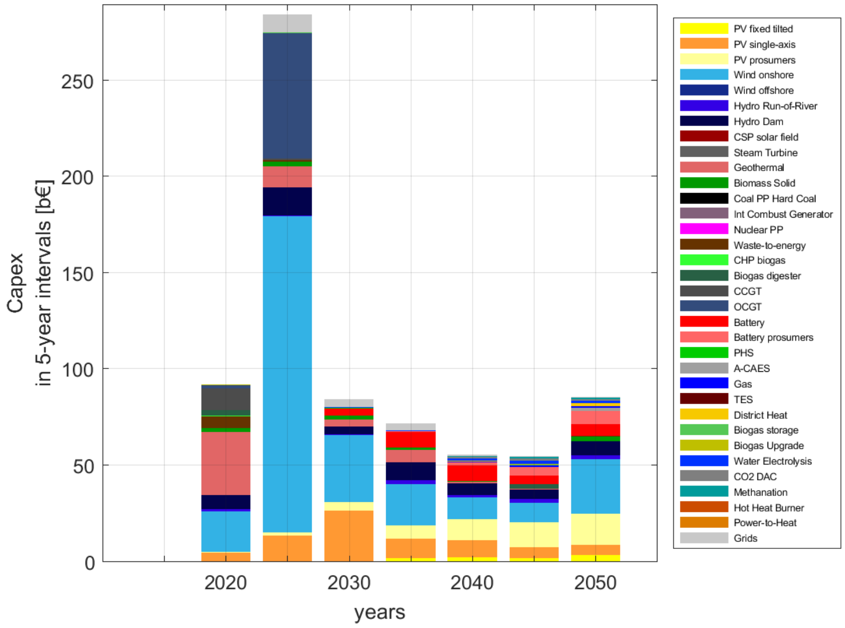

Supplementary Figure 44. Eurasia - CAPEX breakdown by technology during the energy transition from 2015 to 2050.

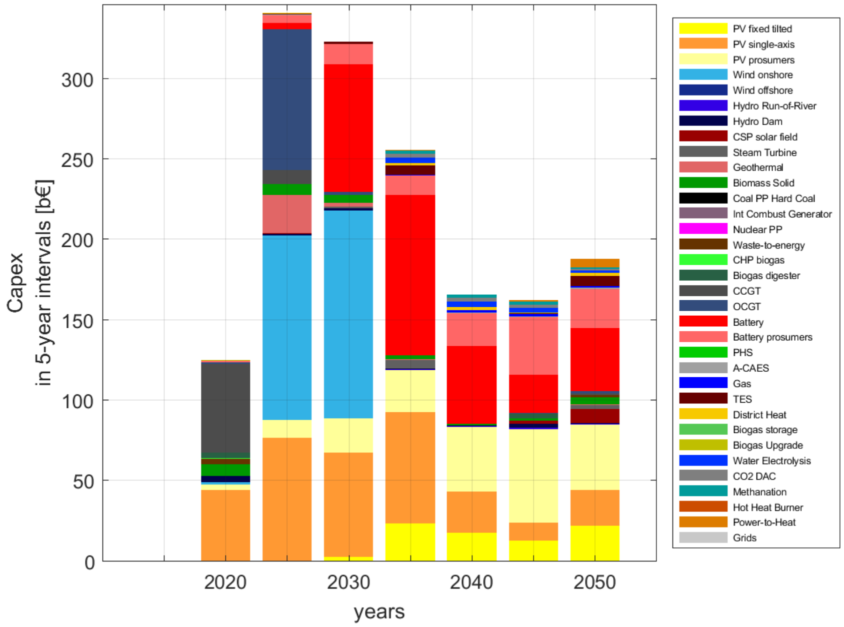

Supplementary Figure 45. MENA - CAPEX breakdown by technology during the energy transition from 2015 to 2050.

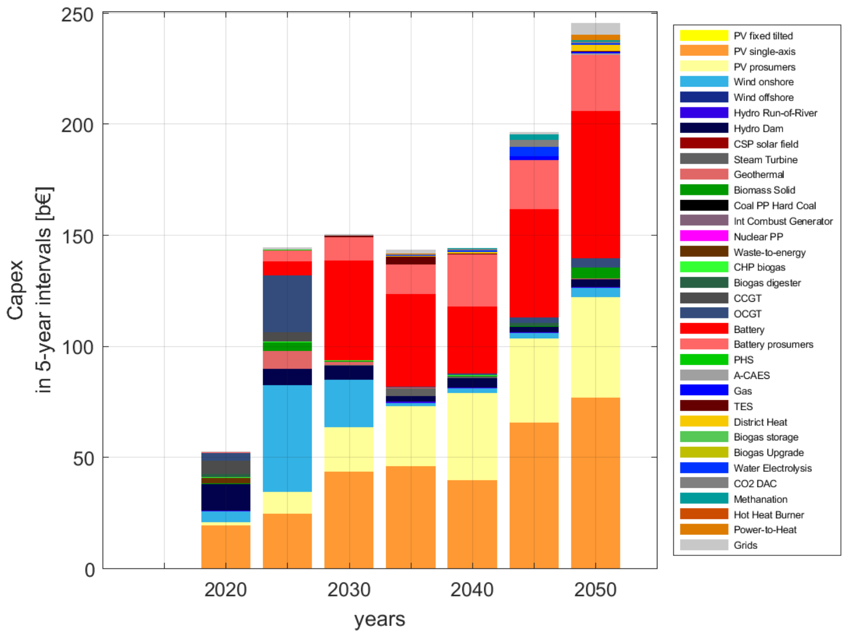

Supplementary Figure 46. Sub-Saharan Africa - CAPEX breakdown by technology during the energy transition from 2015 to 2050.

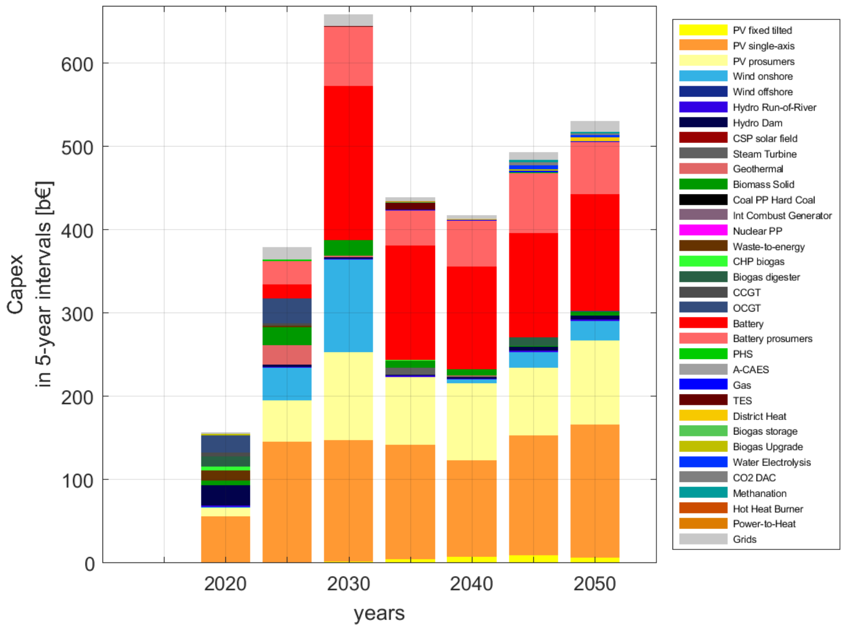

Supplementary Figure 47. SAARC - CAPEX breakdown by technology during the energy transition from 2015 to 2050.

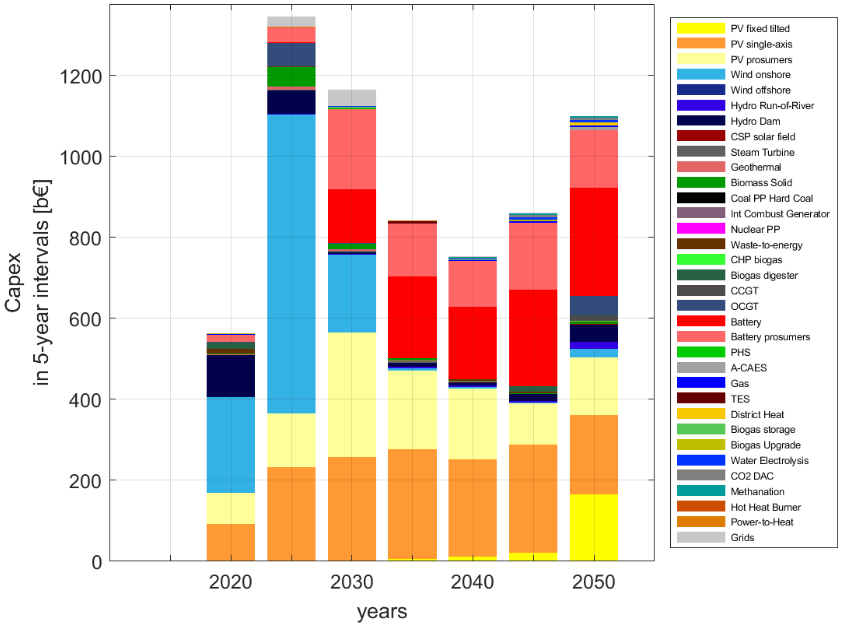

Supplementary Figure 48. Northeast Asia - CAPEX breakdown by technology during the energy transition from 2015 to 2050.

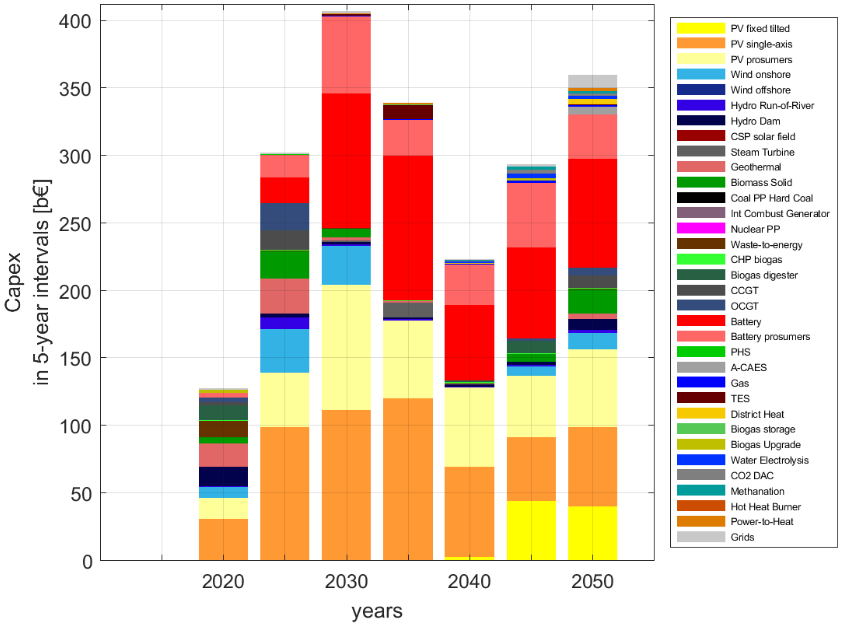

Supplementary Figure 49. Southeast Asia - CAPEX breakdown by technology during the energy transition from 2015 to 2050.

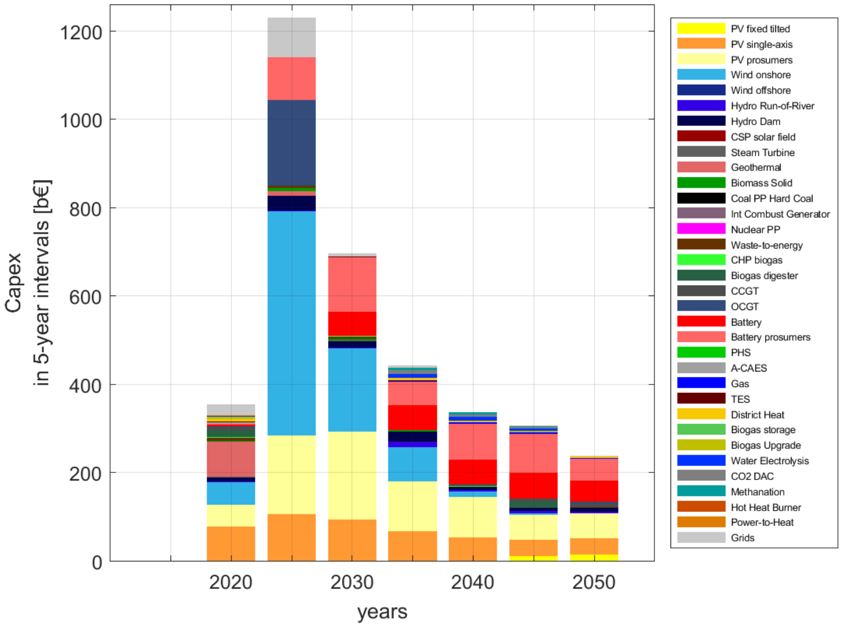

Supplementary Figure 50. North America - CAPEX breakdown by technology during the energy transition from 2015 to 2050.

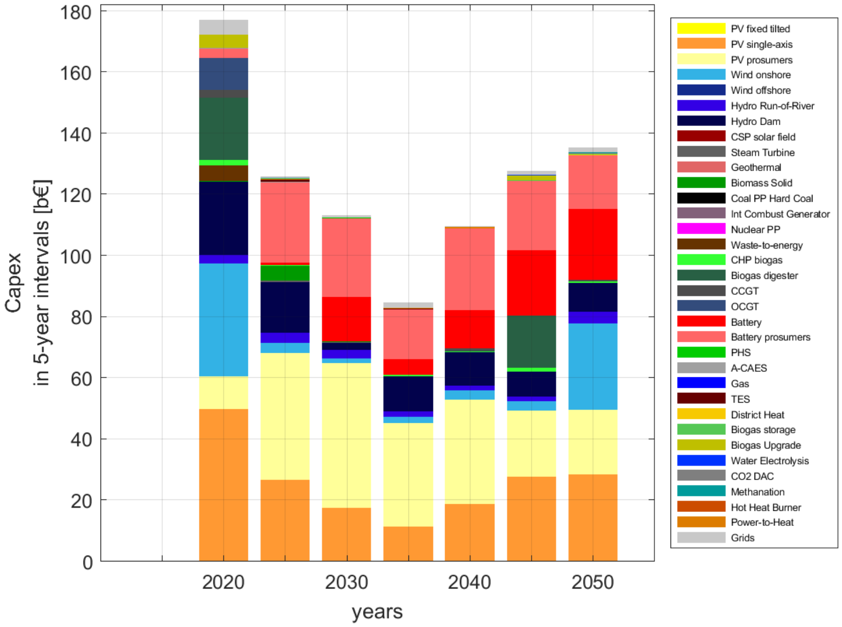

Supplementary Figure 51. South America - CAPEX breakdown by technology during the energy transition from 2015 to 2050.

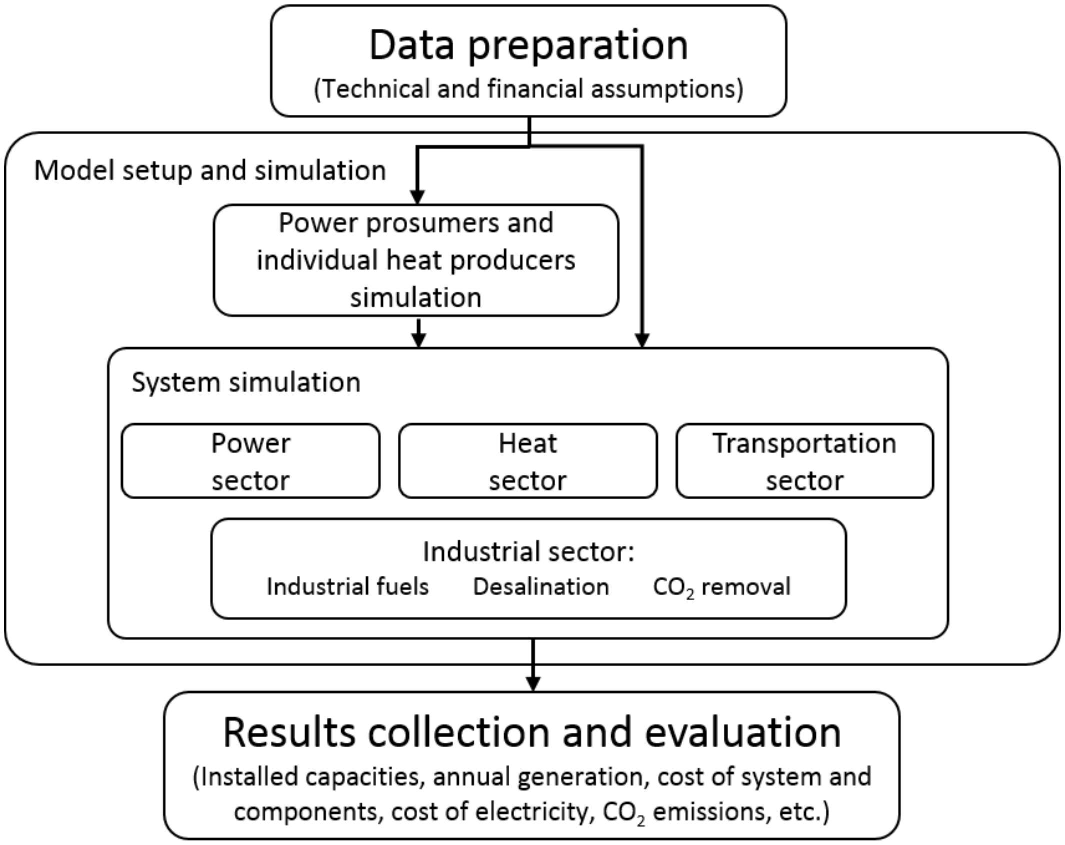

Supplementary Figure 52. The overall structure of the modelling procedure.

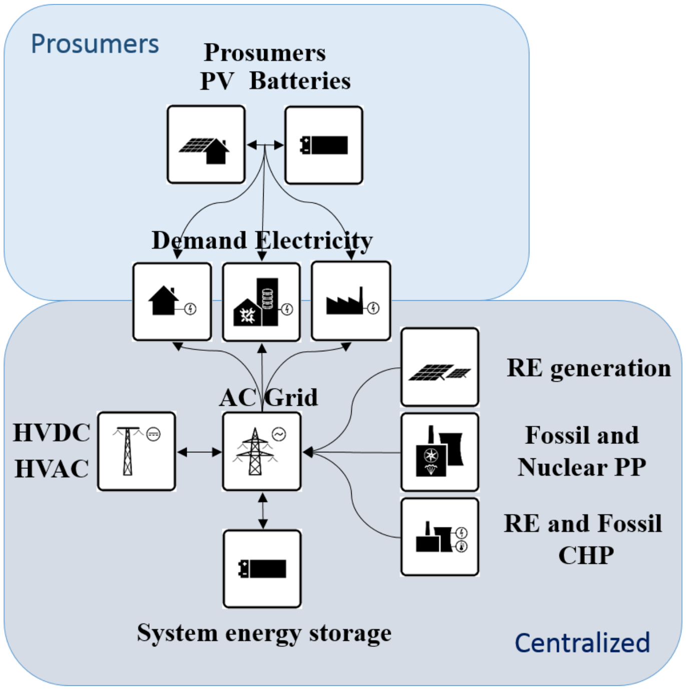

Supplementary Figure 53. Power sector structure.

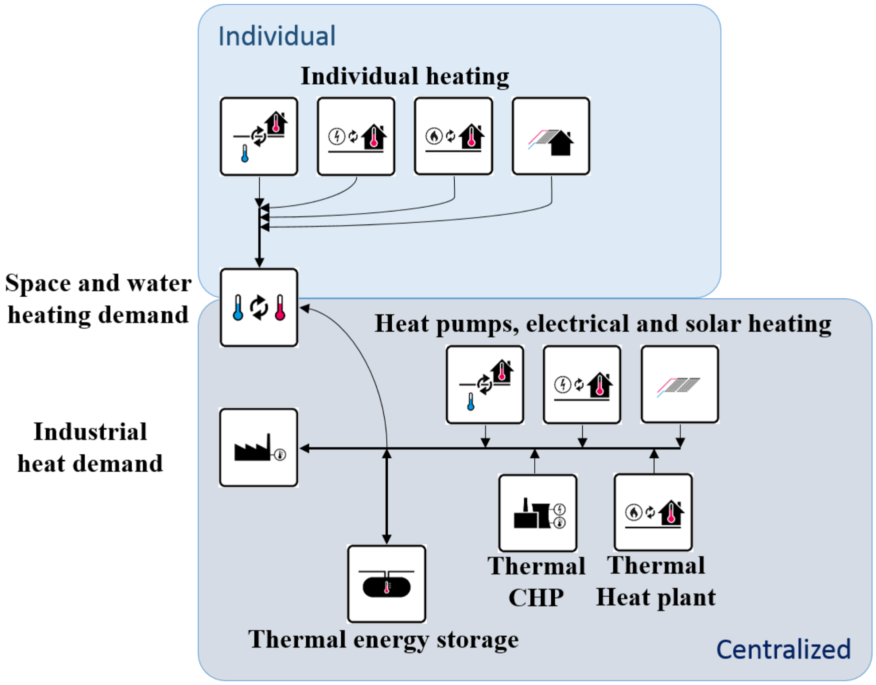

Supplementary Figure 54. Heat sector structure.

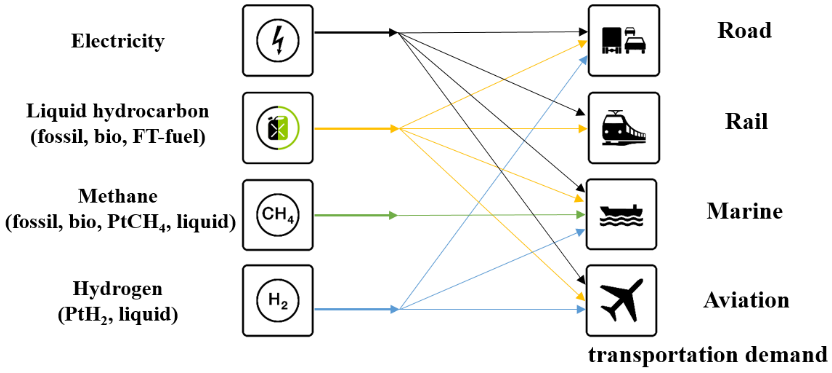

Supplementary Figure 55. Transport sector structure.

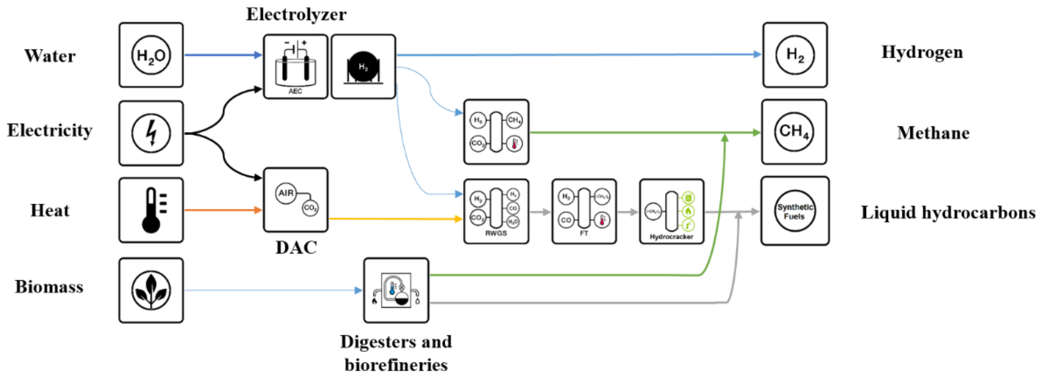

Supplementary Figure 56. Industrial fuels production sector structure.

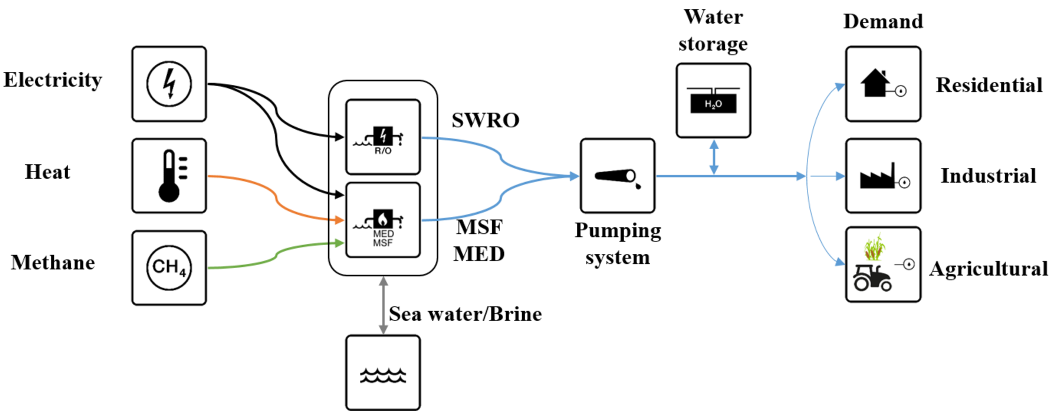

Supplementary Figure 57. Desalination sector structure.

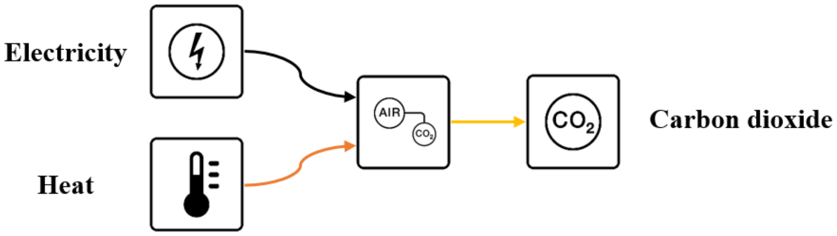

Supplementary Figure 58. CO_2 removal sector structure.

Supplementary Table 1. The list of the regions and their description.

		Abbr.	Countries
EUROPE	**1**	'NO'	Norway
	**2**	'DK'	Denmark
	**3**	'SE'	Sweden
	**4**	'FI'	Finland
	**5**	'BLT'	Baltic: Estonia, Latvia, Lithuania
	**6**	'PL'	Poland
	**7**	'IBE'	Iberia: Portugal, Spain, Gibraltar
	**8**	'FR'	France, Monaco, Andorra
	**9**	'BNL'	Belgium, Netherlands, Luxembourg
	**10**	'BRI'	British Isles: Ireland, United Kingdom, Isle of Man, Guernsey, Jersey
	**11**	'DE'	Germany
	**12**	'CRS'	Czech Republic, Slovakia
	**13**	'AUH'	Austria, Hungary
	**14**	'BKN-W'	Balkan-West: Slovenia, Croatia, Bosnia and Hertzegovina, Serbia, Montenegro, Macedonia, Albania
	**15**	'BKN-E'	Balkan-East: Romania, Bulgaria, Greece
	**16**	'IT'	Italy, San Marino, Vatican, Malta
	**17**	'CH'	Switzerland, Liechtenstein
	**18**	'TR'	Turkey, Cyprus
	**19**	'UA'	Ukraine, Moldova
	**20**	'IS'	Iceland
MENA	**21**	'DZ'	Algeria
	**22**	'BHQ'	Bahrain and Qatar
	**23**	'EG'	Egypt
	**24**	'IR'	Iran
	**25**	'IQ'	Iraq
	**26**	'IL'	Israel
	**27**	'JWG'	Jordan (incl. West Bank & Gaza Strip = State of Palestine)
	**28**	'KW'	Kuwait
	**29**	'LB'	Lebanon
	**30**	'LY'	Libya
	**31**	'MA'	Morocco
	**32**	'OM'	Oman
	**33**	'SA'	Saudi Arabia
	**34**	'TN'	Tunisia
	**35**	'AE'	United Arab Emirates
	**36**	'YE'	Yemen
	**37**	'SY'	Syria

EURASIA	**38**	'RU-NW'	Russia North-West
	**39**	'RU-C'	Russia Center
	**40**	'RU-S'	Russia South
	**41**	'RU-V'	Russia Volga region
	**42**	'RU-U'	Russia Urals
	**43**	'RU-SI'	Russia Siberia
	**44**	'RU-FE'	Russia Far East
	**45**	'BY'	Belarus
	**46**	'CAU'	Armenia, Azerbaijan, Georgia
	**47**	'KZ'	Kazakhstan
	**48**	'PAM'	Tajikistan, Kyrgyzstan
	**49**	'UZ'	Uzbekistan
	**50**	'TM'	Turkmenistan
NORTH-EAST ASIA	**51**	'JP-E'	Japan East
	**52**	'JP-W'	Japan West
	**53**	'KR'	South Korea (Republic of Korea)
	**54**	'KP'	North Korea (DPR of Korea)
	**55**	'CN-NE'	China North-East
	**56**	'CN-N'	China North
	**57**	'CN-E'	China East
	**58**	'CN-C'	China Central
	**59**	'CN-S'	China South
	**60**	'CN-TB'	China Tibet
	**61**	'CN-NW'	China North-Wes)
	**62**	'CN-XU'	China Uyghur region
	**63**	'MN'	Mongolia
SOUTH-EAST ASIA	**64**	'NZ'	New Zealand
	**65**	'AU-E'	Australia East
	**66**	'AU-W'	Australia West
	**67**	'ID-PG+NG'	Indonesia-Papua and Papua New Guinea
	**68**	'ID-SU'	Indonesia Sumatra
	**69**	'ID-JV+TL'	Indonesia Java and Timor Leste
	**70**	'ID-KL-SW'	Indonesia East
	**71**	'MY-W+SG'	Malaysia West and Singapore
	**72**	'MY-E+BN'	Malaysia East and Brunei
	**73**	'PH'	Philippines
	**74**	'MM'	Myanmar
	**75**	'TH'	Thailand
	**76**	'LA'	Laos
	**77**	'VN'	Vietnam
	**78**	'KH'	Cambodia

**SAARC**	**79**	'IN-E'	India East
	**80**	'IN-CE'	India Central-East
	**81**	'IN-W'	India West
	**82**	'IN-CW'	India Central-West
	**83**	'IN-N'	India North
	**84**	'IN-NW'	India North-West
	**85**	'IN-UP'	India Uttar Pradesh
	**86**	'IN-S'	India South
	**87**	'IN-CS'	India South-Central
	**88**	'IN-NE'	India North-East
	**89**	'BD'	Bangladesh
	**90**	'NP+BT'	Nepal and Bhutan
	**91**	'PK-S'	Pakistan South
	**92**	'PK-N'	Pakistan North
	**93**	'AF'	Afghanistan
	**94**	'LK'	Sri Lanka
**SUB-SAHARAN AFRICA**	**95**	'WW'	Senegal, Gambia, Cape Verde Islands, Guinea Bissau, Guinea, Sierra Leone, Liberia, Mali, Mauritania, Western Sahara
	**96**	'WS'	Ghana, Cote D'Ivoire, Benin, Burkina Faso (Upper Volta), Togo
	**97**	'WN'	Niger, Chad
	**98**	'NIG-S'	Nigeria South
	**99**	'NIG-N'	Nigeria North
	**100**	'SER'	Sudan, Eritrea
	**101**	'ETH'	Ethiopia
	**102**	'SOMDJ'	Djibouti, Somalia
	**103**	'KENUG'	Kenya, Uganda
	**104**	'TZRB'	Rwanda, Burundi, Tanzania
	**105**	'CAR'	Central African Republic, Cameroon, Equatorial Guinea, Sao Tome and Principe, Congo, Republic of, Gabon
	**106**	'COG'	Congo, Democratic Republic
	**107**	'SW'	Angola, Namibia, Botswana
	**108**	'ZAFLS'	Republic of South Africa, Lesotho
	**109**	'SE'	Malawi, Mozambique, Zambia, Zimbabwe, Swaziland
	**110**	'IOCE'	Comoros Islands, Mauritius, Mayotte, Madagascar, Seychelles
**SOUTH AMERICA**	**111**	'CAM'	Panama, Costa Rica, Nicaragua, Honduras, El Salvador, Guatemala and Belize
	**112**	'CO'	Colombia
	**113**	'VE'	Venezuela, Guyana, French Guiana, Suriname
	**114**	'EC'	Ecuador
	**115**	'PE'	Peru
	**116**	'CSA'	Bolivia and Paraguay
	**117**	'BR-S'	Brazil South
	**118**	'BR-SP'	Brazil São Paulo

	**119**	'BR-SE'	Brazil Southeast
	**120**	'BR-N'	Brazil North
	**121**	'BR-NE'	Brazil Northeast
	**122**	'AR-NE'	Argentina Northeast, Uruguay
	**123**	'AR-E'	Argentina East
	**124**	'AR-W'	Argentina West
	**125**	'CL'	Chile
**NORTH AMERICA**	**126**	'CA-W'	Canada West
	**127**	'CA-E'	Canada East
	**128**	'US-NENY'	US New England & New York (Connecticut, Maine, Massachusetts, New Hampshire, Rhode Island, Vermont, New York)
	**129**	'US-MA'	US Mid-Atlantics (Delaware, Maryland, New Jersey, Ohio, Pennsylvania, West Virginia, Virginia)
	**130**	'US-CAR'	US Carolinas (North Carolina, South Carolina)
	**131**	'US-S'	US Southern (Alabama, Florida, Georgia)
	**132**	'US-TVA'	US TVA (Kentucky, Tennessee)
	**133**	'US-MW'	US Midwest (Illinois, Indiana, Iowa, Michigan, Minnesota, Missouri, North Dakota, South Dakota, Wisconsin)
	**134**	'US-C'	US Central (Kansas, Nebraska, Oklahoma)
	**135**	'US-TX'	US Texas
	**136**	'US-SW'	US Southwest (Arizona, New Mexico)
	**137**	'US-NW'	US Northwest (Colorado, District of Columbia, Idaho, Montana, Nevada, Oregon, Utah, Washington, Wyoming)
	**138**	'US-CA'	US California
	**139**	'US-AK'	US Alaska
	**140**	'US-HI'	US Hawaii
	**141**	'US-GU'	US Gulf (Arkansas, Louisiana, Mississippi)
	**142**	'MX-NW'	Mexico Northwest
	**143**	'MX-N'	Mexico North
	**144**	'MX-C'	Mexico Centre
	**145**	'MX-S'	Mexico South

Supplementary Table 2. VRE technologies FLh and biomass resources availability.

The datasets for solar irradiation components and wind speed are taken from NASA databases[1,2] and partly reprocessed by the German Aerospace Center[3]. The regional average generation profiles for PV, solar CSP and wind energy are calculated according to Bogdanov and Breyer (2016)[4]. Wind turbines full load hours are calculated for the case on Enercom E-101 turbine at 150m hub height. The hydropower feed-in profiles are computed based on daily resolved water flow data for the year 2005[5]. The potentials for biomass and waste resources were taken from Bunzel et al. (2009)[6] and Elbersen et al. (2012)[7] and classified into three main categories: solid wastes, solid residues and biogas.

	Region	Full load hours				Annual potential [TWh]		
		PV optimally tilted	PV single-axis	Wind onshore	Hydro Run-of-river	Biomass Solid residues	Biomass Solid waste	Biomass Biogas
**1**	'NO'	882	1112	3911	3785	8.5	2.7	1.4
**2**	'DK'	1070	1346	4991	2821	15.2	3.1	28.4
**3**	'SE'	985	1229	2918	3795	48.3	71.7	6.7
**4**	'FI'	986	1288	2930	4162	36.5	60.6	13.2
**5**	'BLT'	1063	1352	3835	1427	24.5	22.6	4.7
**6**	'PL'	1065	1269	3373	1717	65.9	29.6	136.3
**7**	'IBE'	1624	2095	2906	2193	47.3	39.2	83.2
**8**	'FR'	1302	1573	3515	2502	148.0	37.7	136.1
**9**	'BNL'	1030	1230	4318	1380	8.3	16.4	75.0
**10**	'BRI'	956	1124	5128	1448	36.7	27.1	99.4
**11**	'DE'	1053	1226	3721	1467	122.1	75.7	61.1
**12**	'CRS'	1098	1292	2828	1481	37.0	26.0	30.5
**13**	'AUH'	1174	1379	2280	4159	57.0	27.9	34.4
**14**	'BKN-W'	1319	1582	1913	3778	21.4	2.9	5.4
**15**	'BKN-E'	1380	1680	2167	1939	82.6	24.2	40.5
**16**	'IT'	1439	1772	2225	1404	38.6	22.0	78.3
**17**	'CH'	1250	1488	1966	2322	5.6	2.9	2.2
**18**	'TR'	1593	2022	2707	3431	41.3	13.9	6.4
**19**	'UA'	1219	1484	2948	1661	42.9	5.8	6.9
**20**	'IS'	819	1093	5396	6984	0.0	0.1	0.0
**21**	'DZ'	1773	2304	3189	2036	4.9	0.6	3.0
**22**	'BHQ'	1714	2215	2625	0	0.0	0.2	0.2
**23**	'EG'	1866	2447	3064	4664	40.7	1.5	12.7
**24**	'IR'	1789	2297	3049	2421	32.8	4.8	7.4
**25**	'IQ'	1691	2143	2941	3111	9.4	2.1	3.2
**26**	'IL'	1747	2236	1472	0	0.5	0.6	0.6
**27**	'JWG'	1796	2347	2890	6670	0.1	0.4	0.4
**28**	'KW'	1659	2185	3174	0	0.1	0.2	0.2

**29**	'LB'	1671	2143	1624	3335	2.0	0.2	1.8
**30**	'LY'	1851	2397	3097	0	0.3	0.1	0.6
**31**	'MA'	1790	2344	2974	1399	8.4	0.6	3.3
**32**	'OM'	1846	2463	2731	0	0.2	0.2	0.2
**33**	'SA'	1830	2443	2815	0	4.1	1.9	2.0
**34**	'TN'	1677	2072	2880	0	2.5	0.2	1.1
**35**	'AE'	1774	2320	2039	0	0.0	0.3	0.3
**36**	'YE'	1883	2553	2637	0	1.4	1.7	1.3
**37**	'SY'	1688	2137	2978	2771	7.2	1.6	1.1
**38**	'RU-NW'	979	1262	3751	3830	16.3	2.5	1.8
**39**	'RU-C'	1130	1408	3064	2018	8.1	7.0	5.2
**40**	'RU-S'	1241	1511	3255	2507	44.2	4.2	3.2
**41**	'RU-V'	1184	1473	3067	3647	37.4	5.4	4.0
**42**	'RU-U'	1014	1292	3613	4714	23.5	2.2	1.6
**43**	'RU-SI'	1174	1491	3145	3527	64.1	3.5	2.6
**44**	'RU-FE'	1136	1477	2798	3917	57.9	1.1	0.8
**45**	'BY'	1058	1281	2924	4954	7.7	2.8	2.8
**46**	'CAU'	1414	1667	1936	2692	4.7	1.1	2.4
**47**	'KZ'	1427	1815	3248	3337	22.2	0.8	3.0
**48**	'PAM'	1688	2178	2410	7484	4.2	0.6	1.4
**49**	'UZ'	1518	1931	2853	3055	9.1	1.4	3.6
**50**	'TM'	1534	1936	2856	0	4.1	0.3	1.0
**51**	'JP-E'	1316	1536	3469	1682	9.7	11.0	7.7
**52**	'JP-W'	1365	1604	3305	4260	9.5	10.9	7.6
**53**	'KR'	1467	1733	3039	4828	4.6	13.3	2.3
**54**	'KP'	1469	1749	2982	5000	5.6	1.0	2.8
**55**	'CN-NE'	1457	1832	3630	2149	63.4	5.6	12.6
**56**	'CN-N'	1592	2011	3653	2668	150.8	13.4	30.0
**57**	'CN-E'	1340	1549	2149	2481	146.1	13.0	29.1
**58**	'CN-C'	1471	1726	2690	3908	214.6	19.1	42.7
**59**	'CN-S'	1435	1678	2383	3973	142.8	12.7	28.4
**60**	'CN-TB'	1983	2719	5372	6994	1.7	0.2	0.3
**61**	'CN-NW'	1739	2221	3819	3966	43.2	3.9	8.6
**62**	'CN-XU'	1666	2124	2810	3689	12.6	1.1	2.5
**63**	'MN'	1572	2062	3391	1739	0.1	0.1	0.1
**64**	'NZ'	1430	1765	4252	4238	15.1	2.3	9.6
**65**	'AU-E'	1733	2316	3611	1765	50.3	14.2	36.1
**66**	'AU-W'	1764	2397	3902	4773	17.7	2.2	5.6
**67**	'ID-PG+NG'	1465	1816	1219	5619	21.2	0.5	0.5
**68**	'ID-SU'	1445	1746	454	2771	56.6	4.2	5.0

**69**	'ID-JV+TL'	1683	2203	1264	2235	101.8	13.5	16.1
**70**	'ID-KL-SW'	1503	1869	406	3823	51.5	2.7	3.2
**71**	'MY-W+SG'	1485	1835	468	3243	13.9	0.5	0.5
**72**	'MY-E+BN'	1489	1810	175	5355	11.3	2.0	2.1
**73**	'PH'	1503	1929	1856	3346	71.1	5.3	15.4
**74**	'MM'	1539	1843	874	4150	38.9	2.5	4.9
**75**	'TH'	1495	1794	1608	2450	10.2	8.6	26.7
**76**	'LA'	1439	1677	963	4686	4.3	0.5	0.6
**77**	'VN'	1456	1764	1896	4613	59.9	6.1	13.2
**78**	'KH'	1512	1810	1268	3145	8.1	1.2	1.1
**79**	'IN-E'	1535	1870	1303	3704	74.6	5.8	14.6
**80**	'IN-CE'	1595	1995	1659	3356	66.3	5.6	14.1
**81**	'IN-W'	1624	1995	2469	1951	94.4	5.6	14.3
**82**	'IN-CW'	1579	1950	2370	2487	90.2	5.8	14.6
**83**	'IN-N'	1878	2515	2517	4068	38.6	1.2	3.1
**84**	'IN-NW'	1633	2011	1678	3561	90.4	5.8	14.7
**85**	'IN-UP'	1639	2100	1803	2222	97.7	8.3	21.1
**86**	'IN-S'	1536	1946	2243	3036	79.4	6.0	15.1
**87**	'IN-CS'	1559	1937	2507	3864	72.0	4.6	11.7
**88**	'IN-NE'	1525	1808	1029	3872	35.8	1.6	4.0
**89**	'BD'	1516	1843	1187	5565	53.0	7.2	6.9
**90**	'NP+BT'	1789	2334	1629	5147	13.5	1.3	1.8
**91**	'PK-S'	1775	2236	2406	6045	21.0	3.2	2.5
**92**	'PK-N'	1697	2187	1570	4717	39.6	6.0	5.2
**93**	'AF'	1802	2340	3558	2116	0.0	0.0	0.8
**94**	'LK'	1536	2028	4022	3161	8.3	1.6	1.2
**95**	'WW'	1713	2258	3341	4517	2.7	0.4	0.6
**96**	'WS'	1615	2099	1530	4231	2.4	0.6	0.8
**97**	'WN'	1870	2520	3652	4558	3.4	0.3	0.4
**98**	'NIG-S'	1469	1832	766	3120	9.3	0.9	1.1
**99**	'NIG-N'	1697	2197	2129	4300	7.9	0.8	0.9
**100**	'SER'	1811	2363	3591	4153	3.7	0.3	1.4
**101**	'ETH'	1771	2370	3291	4037	12.5	1.5	1.4
**102**	'SOMDJ'	1788	2442	4903	0	1.2	0.0	0.0
**103**	'KENUG'	1735	2408	4131	5662	1.3	0.7	1.1
**104**	'TZRB'	1755	2409	2810	4984	0.0	0.3	0.3
**105**	'CAR'	1589	1992	1199	4452	2.7	0.5	0.7
**106**	'COG'	1554	1917	998	6747	9.1	1.1	1.0
**107**	'SW'	1815	2413	2885	4467	2.4	0.5	0.3

**108**	'ZAFLS'	1822	2456	3398	2705	10.3	2.3	2.6
**109**	'SE'	1730	2286	2742	5127	3.4	1.5	2.3
**110**	'IOCE'	1838	2514	3290	3160	2.6	0.4	1.1
**111**	'CAM'	1633	2141	1727	4594	17.9	4.4	15.5
**112**	'CO'	1520	1835	886	4454	15.5	1.8	15.4
**113**	'VE'	1573	1981	1087	2863	11.7	1.7	7.3
**114**	'EC'	1510	1942	2150	5881	5.2	1.2	3.7
**115**	'PE'	1820	2414	2013	5950	7.7	0.9	4.8
**116**	'CSA'	1775	2341	3120	4314	17.0	1.4	5.1
**117**	'BR-S'	1470	1877	2232	3078	57.7	0.7	24.7
**118**	'BR-SP'	1544	1984	1834	5509	72.5	1.1	37.4
**119**	'BR-SE'	1588	2070	1709	3678	78.4	1.1	34.9
**120**	'BR-N'	1499	1904	913	5207	180.1	0.8	27.5
**121**	'BR-NE'	1668	2296	3739	4559	122.3	1.4	47.8
**122**	'AR-NE'	1497	1957	3191	2916	30.3	0.7	2.3
**123**	'AR-E'	1532	2008	4242	0	57.7	1.2	4.6
**124**	'AR-W'	1799	2425	5326	3853	48.5	0.9	3.3
**125**	'CL'	1909	2641	5006	3952	42.9	4.4	1.8
**126**	'CA-W'	1113	1424	3213	4385	155.7	6.3	7.0
**127**	'CA-E'	1258	1555	4098	5023	71.9	13.9	15.5
**128**	'US-NENY'	1317	1597	2212	4006	26.1	7.1	18.5
**129**	'US-MA'	1353	1616	2633	3007	27.3	10.4	27.1
**130**	'US-CAR'	1456	1754	2367	2731	17.7	3.0	7.9
**131**	'US-S'	1536	1903	2063	2359	36.6	7.1	18.5
**132**	'US-TVA'	1427	1732	2383	4174	17.0	2.3	5.9
**133**	'US-MW'	1408	1755	4068	2764	127.3	10.6	27.7
**134**	'US-C'	1562	1983	4310	3113	47.3	1.8	4.6
**135**	'US-TX'	1698	2168	3871	2059	55.4	5.4	14.1
**136**	'US-SW'	1780	2348	3367	3203	104.4	7.2	18.8
**137**	'US-NW'	1652	2160	3531	3277	164.5	5.3	13.8
**138**	'US-CA'	1758	2347	2261	2173	33.8	7.9	20.6
**139**	'US-AK'	966	1330	3588	3786	135.4	0.2	0.4
**140**	'US-HI'	1812	2468	2980	2864	2.3	0.3	0.8
**141**	'US-GU'	1496	1832	2076	3160	31.6	2.2	5.7
**142**	'MX-NW'	1842	2475	1983	2775	16.2	0.6	2.3
**143**	'MX-N'	1853	2371	2897	2773	22.6	0.9	3.9
**144**	'MX-C'	1837	2419	2783	1754	18.4	1.6	6.5
**145**	'MX-S'	1762	2298	2082.4	3166.7	29.2	3.657503	15.25535

Supplementary Table 3. RE technologies, upper capacity limits.

Upper limits for PV and CSP solar field are based on the assumption that up to 6% of the regional area can be covered by solar fields. For onshore wind only 4% of the area can be used for wind farms due to societal constraints. Area requirements for PV and wind are calculated according to Bogdanov and Breyer[4]. The hydro upper limit is based on the assumption that existing capacities can be expanded by 50%.

	Region	Capacity [GW]				
		PV	CSP solar field	Wind onshore	Hydro reservoir (dam)	Hydro Run-of-river
**1**	'NO'	1457	2914	109	30	14
**2**	'DK'	194	388	14	0	0
**3**	'SE'	2026	4053	151	17	8
**4**	'FI'	1522	3043	114	0	5
**5**	'BLT'	788	1576	59	0	2
**6**	'PL'	1407	2814	105	1	0
**7**	'IBE'	2689	5377	201	27	8
**8**	'FR'	2484	4968	185	19	9
**9**	'BNL'	336	672	25	0	0
**10**	'BRI'	1416	2832	106	2	1
**11**	'DE'	1607	3213	120	3	5
**12**	'CRS'	576	1151	43	4	1
**13**	'AUH'	796	1592	59	7	8
**14**	'BKN-W'	1281	2562	96	9	4
**15**	'BKN-E'	2166	4331	162	13	5
**16**	'IT'	1358	2715	101	14	8
**17**	'CH'	186	371	14	12	6
**18**	'TR'	3568	7135	266	25	11
**19**	'UA'	2868	5737	214	3	4
**20**	'IS'	464	927	35	3	0
**21**	'DZ'	10718	21436	800	0	0
**22**	'BHQ'	56	111	4	0	0
**23**	'EG'	4507	9013	336	3	1
**24**	'IR'	7417	14834	554	11	5
**25**	'IQ'	1972	3945	147	3	1
**26**	'IL'	93	187	7	0	0
**27**	'JWG'	430	860	32	0	0
**28**	'KW'	80	160	6	0	0
**29**	'LB'	47	94	3	0	0
**30**	'LY'	7918	15836	591	0	0
**31**	'MA'	2009	4019	150	1	1

**32**	'OM'	1393	2786	104	0	0
**33**	'SA'	9674	19347	722	0	0
**34**	'TN'	736	1472	55	0	0
**35**	'AE'	376	752	28	0	0
**36**	'YE'	2376	4752	177	0	0
**37**	'SY'	833	1667	62	2	0
**38**	'RU-NW'	7551	15101	564	5	2
**39**	'RU-C'	2938	5875	219	9	4
**40**	'RU-S'	2651	5303	198	9	3
**41**	'RU-V'	4671	9342	349	8	1
**42**	'RU-U'	8050	16100	601	0	0
**43**	'RU-SI'	23017	46033	1719	27	12
**44**	'RU-FE'	27972	55943	2089	4	2
**45**	'BY'	913	1826	68	0	0
**46**	'CAU'	812	1625	61	8	2
**47**	'KZ'	12149	24297	907	3	1
**48**	'PAM'	1500	3000	112	3	2
**49**	'UZ'	1914	3829	143	3	1
**50**	'TM'	2115	4229	158	0	0
**51**	'JP-E'	878	1755	66	9	5
**52**	'JP-W'	806	1611	60	12	7
**53**	'KR'	451	891	34	2	0
**54**	'KP'	542	1044	41	0	0
**55**	'CN-NE'	5701	11772	426	8	4
**56**	'CN-N'	5578	10386	417	61	28
**57**	'CN-E'	2123	4311	159	14	5
**58**	'CN-C'	5837	11511	436	100	34
**59**	'CN-S'	4604	9117	344	78	34
**60**	'CN-TB'	5528	10143	413	0	0
**61**	'CN-NW'	6493	12420	485	20	12
**62**	'CN-XU'	7492	14562	559	3	3
**63**	'MN'	7047	13959	526	0	0
**64**	'NZ'	1197	4070	89	5	3
**65**	'AU-E'	18867	64147	1409	6	4
**66**	'AU-W'	15810	53755	1181	0	0
**67**	'ID-PG+NG'	3969	13495	296	0	0
**68**	'ID-SU'	2160	7344	161	1	1
**69**	'ID-JV+TL'	689	2341	51	6	3
**70**	'ID-KL-SW'	3231	10985	241	0	1

**71**	'MY-W+SG'	594	2020	44	3	1
**72**	'MY-E+BN'	929	3158	69	2	1
**73**	'PH'	1341	4561	100	2	2
**74**	'MM'	3014	10248	225	5	1
**75**	'TH'	2318	7881	173	2	1
**76**	'LA'	1038	3528	78	1	0
**77**	'VN'	1472	5005	110	18	9
**78**	'KH'	822	2794	61	1	0
**79**	'IN-E'	1132	2264	85	4	2
**80**	'IN-CE'	782	1565	58	0	0
**81**	'IN-W'	2289	4577	171	4	1
**82**	'IN-CW'	1993	3986	149	4	1
**83**	'IN-N'	1491	2983	111	9	13
**84**	'IN-NW'	1973	3946	147	4	3
**85**	'IN-UP'	1084	2168	81	1	0
**86**	'IN-S'	1279	2558	96	4	2
**87**	'IN-CS'	1584	3168	118	7	3
**88**	'IN-NE'	1148	2296	86	1	1
**89**	'BD'	664	1328	50	0	0
**90**	'NP+BT'	835	1670	62	1	2
**91**	'PK-S'	2197	4393	164	0	0
**92**	'PK-N'	1774	3548	132	9	2
**93**	'AF'	2935	5870	219	0	0
**94**	'LK'	295	590	22	1	1
**95**	'WW'	14460	28920	1080	0	0
**96**	'WS'	4525	9050	338	3	1
**97**	'WN'	9935	19870	742	0	0
**98**	'NIG-S'	3840	7679	287	0	0
**99**	'NIG-N'	4223	8447	315	2	1
**100**	'SER'	11822	23644	883	2	1
**101**	'ETH'	5072	10144	379	2	0
**102**	'SOMDJ'	2973	5946	222	0	0
**103**	'KENUG'	3684	7368	275	1	0
**104**	'TZRB'	4497	8993	336	0	1
**105**	'CAR'	7817	15634	584	1	1
**106**	'COG'	10554	21109	788	2	0
**107**	'SW'	14013	28027	1046	1	1
**108**	'ZAFLS'	5626	11252	420	1	0
**109**	'SE'	9363	18726	699	5	2
**110**	'IOCE'	2664	5329	199	0	0

**111**	'CAM'	2350	7051	175	4	2
**112**	'CO'	5138	15414	384	10	5
**113**	'VE'	6185	18554	462	10	13
**114**	'EC'	1154	3461	86	2	1
**115**	'PE'	5783	17350	432	3	2
**116**	'CSA'	6774	20322	506	10	4
**117**	'BR-S'	2595	7786	194	30	5
**118**	'BR-SP'	1117	3351	83	17	3
**119**	'BR-SE'	3044	9131	227	15	4
**120**	'BR-N'	24570	73711	1835	29	11
**121**	'BR-NE'	6994	20983	522	16	3
**122**	'AR-NE'	3050	9149	228	6	2
**123**	'AR-E'	1385	4155	103	0	0
**124**	'AR-W'	8870	26610	662	10	1
**125**	'CL'	3406	10219	254	6	4
**126**	'CA-W'	30722	61444	2294	18	16
**127**	'CA-E'	14186	28372	1059	56	27
**128**	'US-NENY'	1474	2948	110	8	4
**129**	'US-MA'	1541	3082	115	6	4
**130**	'US-CAR'	1000	2001	75	3	1
**131**	'US-S'	2070	4140	155	5	3
**132**	'US-TVA'	962	1924	72	3	2
**133**	'US-MW'	7196	14392	537	5	2
**134**	'US-C'	2675	5351	200	1	0
**135**	'US-TX'	3131	6261	234	1	0
**136**	'US-SW'	5900	11800	441	3	1
**137**	'US-NW'	9298	18596	694	30	25
**138**	'US-CA'	1908	3816	142	12	5
**139**	'US-AK'	7651	15301	571	0	0
**140**	'US-HI'	127	255	10	0	0
**141**	'US-GU'	1789	3577	134	1	1
**142**	'MX-NW'	1430	2860	107	1	0
**143**	'MX-N'	1998	3997	149	0	0
**144**	'MX-C'	1598	3195	119	4	1
**145**	'MX-S'	2506	5011	187	8	4

Supplementary Table 4. Projected electricity consumption.

Electricity consumption values (for the Power sector structure as of 2015, i.e. without additional demand from other sectors) are mainly based on IEA World Energy Outlook 2015[8] assumptions, and local sources. Electricity demand is calculated with the future transmission and distribution loss projections according to Sadovskaia et al.[9] and the annual electricity demand is distributed for all hours of a year according to the method presented in Toktarova et al.[10].

	Region	Annual electricity consumption [TWh]							
		2015	2020	2025	2030	2035	2040	2045	2050
**1**	'NO'	115	122	129	137	146	155	164	174
**2**	'DK'	32	30	31	32	34	36	39	42
**3**	'SE'	145	155	160	163	174	179	190	199
**4**	'FI'	74	74	85	93	97	101	108	113
**5**	'BLT'	26	26	28	33	33	35	36	38
**6**	'PL'	152	173	181	186	193	202	216	221
**7**	'IBE'	327	340	364	384	403	416	433	443
**8**	'FR'	529	525	545	568	582	600	634	645
**9**	'BNL'	206	205	195	197	203	216	225	231
**10**	'BRI'	356	340	351	365	383	414	438	448
**11**	'DE'	544	533	531	540	544	557	563	580
**12**	'CRS'	95	100	104	110	121	126	131	135
**13**	'AUH'	93	94	100	106	108	113	119	124
**14**	'BKN-W'	88	92	96	101	106	113	117	123
**15**	'BKN-E'	148	156	160	170	180	190	201	208
**16**	'IT'	289	290	302	315	333	359	391	399
**17**	'CH'	61	65	69	73	78	82	87	93
**18**	'TR'	274	382	425	469	520	573	611	649
**19**	'UA'	148	157	166	177	187	199	211	224
**20**	'IS'	17	18	19	20	22	23	24	26
**21**	'DZ'	46	54	62	72	84	97	112	130
**22**	'BHQ'	67	76	87	99	113	129	148	169
**23**	'EG'	146	169	196	227	264	306	354	411
**24**	'IR'	231	264	302	345	394	450	514	588
**25**	'IQ'	55	63	72	82	93	107	122	139
**26**	'IL'	51	58	66	75	86	98	113	129
**27**	'JWG'	16	18	21	24	27	31	35	40
**28**	'KW'	48	55	63	72	82	93	107	122
**29**	'LB'	15	17	19	22	25	28	32	37
**30**	'LY'	32	37	43	50	58	67	78	91
**31**	'MA'	31	36	42	49	57	66	76	89
**32**	'OM'	16	18	21	23	27	31	35	40

**33**	'SA'	289	330	377	431	492	563	643	734
**34**	'TN'	15	17	20	23	27	31	36	42
**35**	'AE'	100	114	131	149	170	195	222	254
**36**	'YE'	6	6	7	8	10	11	13	14
**37**	'SY'	37	42	48	55	62	71	81	93
**38**	'RU-NW'	79	85	90	96	103	110	117	125
**39**	'RU-C'	151	161	172	183	195	208	222	237
**40**	'RU-S'	62	66	71	75	80	86	92	98
**41**	'RU-V'	143	152	162	173	185	197	210	224
**42**	'RU-U'	136	145	154	164	175	187	200	213
**43**	'RU-SI'	166	177	189	202	215	229	245	261
**44**	'RU-FE'	33	35	37	40	43	45	48	52
**45**	'BY'	32	34	36	39	42	45	48	52
**46**	'CAU'	30	32	34	37	39	42	45	49
**47**	'KZ'	71	76	82	88	94	101	108	116
**48**	'PAM'	24	26	28	30	32	34	37	40
**49**	'UZ'	45	49	52	56	60	64	69	74
**50**	'TM'	10	11	12	12	13	14	15	16
**51**	'JP-E'	481	490	500	510	521	531	542	553
**52**	'JP-W'	475	484	494	504	514	524	535	546
**53**	'KR'	490	508	526	544	564	584	604	626
**54**	'KP'	14	16	19	23	27	31	37	43
**55**	'CN-NE'	396	455	522	600	688	790	907	1040
**56**	'CN-N'	943	1080	1240	1430	1640	1880	2160	2480
**57**	'CN-E'	914	1050	1200	1380	1590	1820	2090	2400
**58**	'CN-C'	1340	1540	1770	2030	2330	2680	3070	3530
**59**	'CN-S'	893	1030	1180	1350	1550	1780	2040	2350
**60**	'CN-TB'	270	310	356	409	470	539	619	711
**61**	'CN-NW'	11	13	14	16	19	22	25	29
**62**	'CN-XU'	79	91	104	119	137	157	181	207
**63**	'MN'	5	6	7	9	10	12	14	17
**64**	'NZ'	39	41	42	44	45	47	48	50
**65**	'AU-E'	210	217	225	233	241	250	258	268
**66**	'AU-W'	41	42	43	45	47	48	50	52
**67**	'ID-PG+NG'	5	6	7	9	11	13	15	19
**68**	'ID-SU'	31	37	45	55	66	80	97	118
**69**	'ID-JV+TL'	158	191	231	280	339	410	497	602
**70**	'ID-KL-SW'	17	21	25	30	37	45	54	65
**71**	'MY-W+SG'	161	195	236	285	345	418	506	613

**72**	'MY-E+BN'	24	29	36	43	52	63	76	93
**73**	'PH'	80	97	118	142	173	209	253	306
**74**	'MM'	12	14	17	21	26	31	37	45
**75**	'TH'	175	212	257	311	377	456	552	668
**76**	'LA'	5	6	7	8	10	12	15	18
**77**	'VN'	142	234	344	505	611	740	896	1080
**78**	'KH'	4	5	6	8	9	11	14	16
**79**	'IN-E'	89	113	143	182	231	293	373	474
**80**	'IN-CE'	43	54	69	87	111	141	179	228
**81**	'IN-W'	172	219	278	353	449	570	724	920
**82**	'IN-CW'	174	222	281	357	454	577	732	930
**83**	'IN-N'	38	48	61	78	99	125	159	202
**84**	'IN-NW'	195	247	314	399	507	643	817	1038
**85**	'IN-UP'	104	132	167	213	270	343	436	554
**86**	'IN-S'	122	155	197	251	319	405	514	653
**87**	'IN-CS'	164	208	264	336	427	542	688	874
**88**	'IN-NE'	15	19	24	30	39	49	62	79
**89**	'BD'	54	63	74	86	101	118	138	162
**90**	'NP+BT'	10	11	13	16	18	21	25	29
**91**	'PK-S'	44	51	60	70	82	96	112	131
**92**	'PK-N'	116	136	159	187	219	256	299	351
**93**	'AF'	5	6	7	8	9	11	13	15
**94**	'LK'	12	13	16	18	22	25	30	35
**95**	'WW'	9	12	17	24	34	47	67	94
**96**	'WS'	21	29	40	56	78	108	150	209
**97**	'WN'	1	2	3	4	6	8	12	16
**98**	'NIG-S'	23	33	48	72	100	143	210	304
**99**	'NIG-N'	24	35	50	75	104	148	218	316
**100**	'SER'	9	13	18	26	37	52	73	103
**101**	'ETH'	7	10	14	20	29	42	60	87
**102**	'SOMDJ'	1	1	2	2	3	4	6	9
**103**	'KENUG'	12	17	24	34	49	68	96	136
**104**	'TZRB'	7	10	14	19	27	38	54	76
**105**	'CAR'	10	14	18	24	32	42	55	73
**106**	'COG'	9	13	19	27	37	53	74	105
**107**	'SW'	15	20	27	35	47	61	81	107
**108**	'ZAFLS'	226	250	276	301	338	369	415	460
**109**	'SE'	37	53	75	106	150	213	303	430
**110**	'IOCE'	6	9	13	17	25	35	50	70
**111**	'CAM'	48	55	61	69	78	88	99	111

**112**	'CO'	62	70	79	89	100	113	127	143
**113**	'VE'	96	108	122	137	155	174	196	221
**114**	'EC'	21	24	27	30	34	39	43	49
**115**	'PE'	40	45	50	57	64	72	81	91
**116**	'CSA'	18	20	23	26	29	33	37	41
**117**	'BR-S'	89	100	112	126	142	160	180	203
**118**	'BR-SP'	150	169	190	214	241	271	306	344
**119**	'BR-SE'	114	129	145	163	184	207	233	262
**120**	'BR-N'	69	78	88	99	111	125	141	159
**121**	'BR-NE'	88	99	112	126	141	159	179	202
**122**	'AR-NE'	37	41	46	52	59	66	74	84
**123**	'AR-E'	70	79	88	100	112	126	142	160
**124**	'AR-W'	42	48	54	61	68	77	87	97
**125**	'CL'	71	74	77	80	83	86	90	93
**126**	'CA-W'	165	173	181	189	198	207	216	226
**127**	'CA-E'	325	340	355	372	389	407	425	445
**128**	'US-NENY'	270	282	295	309	323	337	353	369
**129**	'US-MA'	594	622	650	680	711	744	778	813
**130**	'US-CAR'	217	227	237	248	259	271	284	296
**131**	'US-S'	456	477	499	522	546	571	597	625
**132**	'US-TVA'	181	189	198	207	216	226	236	247
**133**	'US-MW'	658	688	719	752	787	823	860	900
**134**	'US-C'	134	140	146	153	160	167	175	183
**135**	'US-TX'	393	411	430	450	470	492	514	538
**136**	'US-SW'	100	105	110	115	120	125	131	137
**137**	'US-NW'	327	342	357	374	391	409	427	447
**138**	'US-CA'	265	277	290	303	317	331	347	363
**139**	'US-AK'	6	7	7	7	7	8	8	9
**140**	'US-HI'	10	10	10	11	11	12	13	13
**141**	'US-GU'	189	197	206	216	226	236	247	258
**142**	'MX-NW'	100	105	110	115	120	126	131	137
**143**	'MX-N'	280	292	306	320	334	350	366	382
**144**	'MX-C'	143	149	156	163	171	179	187	195
**145**	'MX-S'	151	158	165	173	181	189	198	207

Supplementary Table 5. Financial and technical assumptions.

Assumptions are mainly taken from Pleßmann et al.[11] and the European Commission[12], and further references are individually mentioned. All technical and financial assumptions are given in currency values of the year 2015.

**Technologies**		**Units**	**2015**	**2020**	**2025**	**2030**	**2035**	**2040**	**2045**	**2050**	**Ref**
**PV rooftop - residential**	Capex	€/kW$_{,el}$	1360	1169	966	826	725	650	589	537	*(13)*
	Opex fix	€/(kW$_{,el}$ a)	20	17.6	15.7	14.2	12.8	11.7	10.7	9.8	
	Opex var	€/(kWh$_{,el}$)	0	0	0	0	0	0	0	0	
	Lifetime	years	30	30	35	35	35	40	40	40	
**PV rooftop - commercial**	Capex	€/kW$_{,el}$	1360	907	737	623	542	484	437	397	*(13)*
	Opex fix	€/(kW$_{,el}$ a)	20	17.6	15.7	14.2	12.8	11.7	10.7	9.8	
	Opex var	€/(kWh$_{,el}$)	0	0	0	0	0	0	0	0	
	Lifetime	years	30	30	35	35	35	40	40	40	
**PV rooftop - industrial**	Capex	€/kW$_{,el}$	1360	682	548	459	397	353	318	289	*(13)*
	Opex fix	€/(kW$_{,el}$ a)	20	17,6	15,7	14,2	12,8	11,7	10.7	9.8	
	Opex var	€/(kWh$_{,el}$)	0	0	0	0	0	0	0	0	
	Lifetime	years	30	30	35	35	35	40	40	40	
**PV optimally tilted**	Capex	€/kW$_{,el}$	1000	580	466	390	337	300	270	246	*(13)*
	Opex fix	€/(kW$_{,el}$ a)	15	13.2	11.8	10.6	9.6	8.8	8.0	7.4	
	Opex var	€/(kWh$_{,el}$)	0	0	0	0	0	0	0	0	
	Lifetime	years	30	30	35	35	35	40	40	40	
**PV single-axis tracking**	Capex	€/kW$_{,el}$	1150	638	513	429	371	330	297	271	*(13,14)*
	Opex fix	€/(kW$_{,el}$ a)	17.3	15.0	13.0	12.0	11.0	10.0	9.0	8.0	
	Opex var	€/(kWh$_{,el}$)	0	0	0	0	0	0	0	0	
	Lifetime	years	30	30	35	35	35	40	40	40	
**Wind onshore**	Capex	€/kW$_{,el}$	1250	1150	1060	1000	965	940	915	900	*(15)*
	Opex fix	€/(kW$_{,el}$ a)	25	23	21	20	19	19	18	18	
	Opex var	€/(kWh$_{,el}$)	0	0	0	0	0	0	0	0	
	Lifetime	years	25	25	25	25	25	25	25	25	
**Wind offshore**	Capex	€/kW$_{,el}$	3220	2880	2700	2580	2460	2380	2320	2280	
	Opex fix	€/(kW$_{,el}$ a)	113	92	84	77	71	67	58	52	
	Opex var	€/(kWh$_{,el}$)	0	0	0	0	0	0	0	0	
	Lifetime	years	20	25	25	25	25	25	25	25	
**CSP (solar field, parabolic trough)**	Capex	€/kW$_{,th}$	547.8	427.8	369.2	326.9	304	283.6	265.4	249.5	*(16,17)*
	Opex fix	€/(kW$_{,th}$ a)	12.6	9.8	8.5	7.5	7	6.5	6.1	5.7	
	Opex var	€/(kWh$_{,th}$)	0	0	0	0	0	0	0	0	
	Lifetime	years	25	25	25	25	30	30	30	30	
**Geothermal power**	Capex	€/kW$_{,el}$	5250	4970	4720	4470	4245	4020	3815	3610	*(18,12)*
	Opex fix	€/(kW$_{,el}$ a)	80.0	80.0	80.0	80.0	80.0	80.0	80.0	80.0	
	Opex var	€/(kWh$_{,el}$)	0	0	0	0	0	0	0	0	
	Efficiency	%	23.9	23.9	23.9	23.9	23.9	23.9	23.9	23.9	
	Lifetime	years	40	40	40	40	40	40	40	40	
**Water electrolysis**	Capex	€/kW$_{,H2}$	800	685	500	363	325	296	267	248	*(19,20)*
	Opex fix	€/(kW$_{,H2}$ a)	32	27	20	12.7	11.4	10.4	9.4	8.7	
	Opex var	€/(kWh$_{,H2}$)	0.0012	0.0012	0.0012	0.0012	0.0012	0.0012	0.0012	0.0012	
	Efficiency	%	84	84	84	84	84	84	84	84	
	Lifetime	years	30	30	30	30	30	30	30	30	
**Methanation**	Capex	€/kW$_{,CH4}$	492	421	310	278	247	226	204	190	*(19,20)*
	Opex fix	€/(kW$_{,CH4}$ a)	19.7	16.8	12.4	11.1	9.9	9.0	8.2	7.6	
	Opex var	€/(kWh$_{,CH4}$)	0.0015	0.0015	0.0015	0.0015	0.0015	0.0015	0.0015	0.0015	
	Efficiency	%	77	77	77	77	77	77	77	77	
	Lifetime	years	30	30	30	30	30	30	30	30	
**CO_2 direct air capture**	Capex	€/t$_{CO2}$ a	480	411	301	228	201	183	165	154	
	Opex fix	€/t$_{CO2}$ a	19.2	16.4	12.0	9.1	8.0	7.3	6.6	6.1	
	Opex var	€/t$_{CO2}$	7.2	7.2	7.2	7.2	7.2	7.2	7.2	7.2	
	El. cons	kWh$_{,el}$/t$_{CO2}$	250	242	236	225	217.5	210	205	200	
	Heat cons	kWh$_{,th}$/t$_{CO2}$	1750	1670	1590	1500	1425	1350	1300	1250	
	Lifetime	years	30	30	30	30	30	30	30	30	

**Coal PP**	Capex	€/(kW$_{,el}$)	1500	1500	1500	1500	1500	1500	1500	1500	
	Opex fix	€/(kW$_{el}$ a)	20	20	20	20	20	20	20	20	
	Opex var	€/(kWh)	0.001	0.001	0.001	0.001	0.001	0.001	0.001	0.001	
	Efficiency	%	43	43	43	43	43	43	43	43	
	Lifetime	years	40	40	40	40	40	40	40	40	
**ICG**	Capex	€/(kW$_{el}$)	310	310	310	310	310	310	310	310	
	Opex fix	€/(kW$_{el}$ a)	6.2	6.2	6.2	6.2	6.2	6.2	6.2	6.2	
	Opex var	€/(kWh$_{,el}$)	0	0	0	0	0	0	0	0	
	Efficiency	%	30	30	30	30	30	30	30	30	
	Lifetime	years	20	20	20	20	20	20	20	20	
**Nuclear PP**	Capex	€/(kW$_{el}$)	6210	6003	6003	5658	5658	5244	5244	5175	
	Opex fix	€/(kW$_{el}$ a)	162	157	157	137	137	116	116	109	
	Opex var	€/(kWh$_{,el}$)	0.0025	0.0025	0.0025	0.0025	0.0025	0.0025	0.0025	0.0025	
	Efficiency	%	37	37	37	38	38	38	38	38	
	Lifetime	years	40	40	40	40	40	40	40	40	
**CCGT**	Capex	€/(kW$_{el}$)	775	775	775	775	775	775	775	775	
	Opex fix	€/(kW$_{el}$ a)	19.4	19.4	19.4	19.4	19.4	19.4	19.4	19.4	
	Opex var	€/(kWh$_{,el}$)	0	0	0	0	0	0	0	0	
	Efficiency	%	58	58	58	58	59	60	60	60	
	Lifetime	years	35	35	35	35	35	35	35	35	
**OCGT**	Capex	€/(kW$_{el}$)	475	475	475	475	475	475	475	475	
	Opex fix	€/(kW$_{el}$ a)	14.25	14.25	14.25	14.25	14.25	14.25	14.25	14.25	
	Opex var	€/(kWh$_{,el}$)	0	0	0	0	0	0	0	0	
	Efficiency	%	43	43	43	43	43	43	43	43	
	Lifetime	years	35	35	35	35	35	35	35	35	
**Steam turbine (CSP)**	Capex	€/(kW$_{el}$)	760	740	720	700	670	640	615	600	
	Opex fix	€/(kW$_{el}$ a)	15.2	14.8	14.4	14	13.4	12.8	12.3	12	
	Opex var	€/(kWh$_{,el}$)	0	0	0	0	0	0	0	0	
	Efficiency	%	42	42	42	43	44	44	45	45	
	Lifetime	years	25	25	25	25	30	30	30	30	
**Biomass CHP**	Capex	€/kW$_{,el}$	2755	2620	2475	2330	2195	2060	1945	1830	
	Opex fix	€/(kW$_{,el}$ a)	55.4	47.2	44.6	41.9	39.5	37.1	35	32.9	
	Opex var	€/(kWh$_{,el}$)	0.004	0.004	0.004	0.004	0.004	0.004	0.004	0.004	
	Efficiency	%	35	36	36.5	37	37.5	38	38.5	39	
	Lifetime	years	25	25	25	25	25	25	25	25	
**Biogas CHP**	Capex	€/kW$_{,el}$	503	429	400	370	340	326	311	296	
	Opex fix	€/(kW$_{,el}$ a)	20.1	17.2	16.0	14.8	13.6	13.0	12.4	11.8	
	Opex var	€/(kWh$_{,el}$)	0.001	0.001	0.001	0.001	0.001	0.001	0.001	0.001	
	Efficiency	%	33.5	34.4	37.2	40.0	41.9	43.7	44.2	44.7	
	Lifetime	years	30	30	30	30	30	30	30	30	
**Waste incinerator**	Capex	€/kW$_{,el}$	5940	5630	5440	5240	5030	4870	4690	4540	
	Opex fix	€/(kW$_{,el}$ a)	267.3	253.35	244.8	235.8	226.35	219.15	211.05	204.3	
	Opex var	€/(kWh$_{,el}$)	0.0069	0.0069	0.0069	0.0069	0.0069	0.0069	0.0069	0.0069	
	Efficiency	%	27	31	32.5	34	35.5	37	37	42	
	Lifetime	years	30	30	30	30	30	30	30	30	
**Biogas digester**	Capex	€/kW$_{,th}$	771	731	706	680	653	632	609	589	
	Opex fix	€/(kW$_{,th}$ a)	30.8	29.2	28.2	27.2	26.1	25.3	24.3	23.6	
	Opex var	€/(kWh$_{,th}$)	0	0	0	0	0	0	0	0	
	Efficiency	%	100	100	100	100	100	100	100	100	
	Lifetime	years	20	20	20	20	25	25	25	25	
**Biogas upgrade**	Capex	€/kW$_{,th}$	340	290	270	250	230	220	210	200	*(21)*
	Opex fix	€/(kW$_{,th}$ a)	27.2	23.2	21.6	20	18.4	17.6	16.8	16	
	Opex var	€/(kWh$_{,th}$)	0	0	0	0	0	0	0	0	
	Efficiency	%	98	98	98	98	98	98	98	98	
	Lifetime	years	20	20	20	20	25	25	25	25	
**Battery, Li-ion**	Capex	€/(kWh$_{el}$)	600	300	200	150	120	100	85	75	*(22,23)*
	Opex fix	€/(kWh$_{el}$ a)	24	9	5	3.75	3	2.5	2.125	1.875	
	Opex var	€/(kWh$_{,el}$)	0.0002	0.0002	0.0002	0.0002	0.0002	0.0002	0.0002	0.0002	
	Efficiency	%	90	91	92	93	94	95	95	95	
	Lifetime	years	15	20	20	20	20	20	20	20	
	Lifetime prosumers	years	10	10	10	10	10	10	10	10	

**Adiabatic compressed air energy storage (A-CAES)**	Capex	€/kWh,$_{el}$	35.0	35.0	33.0	31.1	30.4	29.8	28.0	26.3	
	Opex fix	€/(kWh,$_{el}$ a)	0.46	0.46	0.43	0.40	0.40	0.39	0.36	0.34	
	Opex var	€/(kWh,$_{el}$)	0.0012	0.0012	0.0012	0.0012	0.0012	0.0012	0.0012	0.0012	
	Efficiency	%	54	59	65	70	70	70	70	70	
	Lifetime	years	40	55	55	55	55	55	55	55	
**Gas storage**	Capex	€/kWh,$_{th}$	0.05	0.05	0.05	0.05	0.05	0.05	0.05	0.05	
	Opex fix	€/(kWh a)	0.001	0.001	0.001	0.001	0.001	0.001	0.001	0.001	
	Opex var	€/(kWh)	0	0	0	0	0	0	0	0	
	Efficiency	%	100	100	100	100	100	100	100	100	
	Lifetime	years	50	50	50	50	50	50	50	50	
**Pumped hydro storage (PHS)**	Capex	€/kWh,$_{el}$	70	70	70	70	70	70	70	70	
	Opex fix	€/(kWh a)	11	11	11	11	11	11	11	11	
	Opex var	€/(kWh)	0.0002	0.0002	0.0002	0.0002	0.0002	0.0002	0.0002	0.0002	
	Efficiency	%	85	85	85	85	85	85	85	85	
	Lifetime	years	50	50	50	50	50	50	50	50	
**Thermal energy storage (TES)**	Capex	€/kWh,$_{th}$	50	40	30	30	20	20	20	20	
	Opex fix	€/(kWh a)	0.75	0.6	0.45	0.45	0.3	0.3	0.3	0.3	
	Opex var	€/(kWh)	0	0	0	0	0	0	0	0	
	Efficiency	%	90	90	90	90	90	90	90	90	
	Lifetime	years	25	25	25	25	30	30	30	30	
**High voltage alternating current transmission line (HVAC)**	Capex	€/(kW km)	0.458	0.458	0.458	0.458	0.458	0.458	0.458	0.458	
	Opex fix	€/(kW km)	0.003	0.003	0.003	0.003	0.003	0.003	0.003	0.003	
	Opex var	€/(kWh km)	0	0	0	0	0	0	0	0	
	Lifetime	years	50	50	50	50	50	50	50	50	
**High voltage direct current transmission line (HVDC)**	Capex	€/(kW km)	1.044	1.044	1.044	1.044	1.044	1.044	1.044	1.044	*(29)*
	Opex fix	€/(kW km)	0.003	0.003	0.003	0.003	0.003	0.003	0.003	0.003	
	Opex var	€/(kWh km)	0	0	0	0	0	0	0	0	
	Lifetime	years	50	50	50	50	50	50	50	50	
**HVDC Converter pair**	Capex	€/kW	180	180	180	180	180	180	180	180	*(29)*
	Opex fix	€/(kW a)	1.8	1.8	1.8	1.8	1.8	1.8	1.8	1.8	
	Opex var	€/(kWh)	0	0	0	0	0	0	0	0	
	Lifetime	years	50	50	50	50	50	50	50	50	

Supplementary Table 6. Storage technologies technical assumptions.

Energy to Power ratio and self-discharge rates

Technology	Energy/Power ratio [h]	Self-discharge [%/h]	References
**Battery**	6	0	*(22,23)*
**PHS**	8	0	*(12)*
**A-CAES**	100	0.1	*(12)*
**TES**	8	0.2	*(11)*
**Gas storage**	80·24	0	*(11)*

Supplementary Table 7. Financial assumptions for fossil-nuclear fuel prices and GHG emissions.

The referenced values are all till 2040 and are kept stable for later periods (fuels). It needs to be commented that the applied CO_2 price seems to be very moderate for the periods beyond 2040 given CO_2 price results of major IAMs in the order of 1000 USD/t_{CO2eq} around 2050, or even higher[24].

Name of component	*Unit*	*2015*	*2020*	*2025*	*2030*	*2035*	*2040*	*2045*	*2050*	*References*
Coal	€/MWh_{th}	7.7	7.7	8.4	9.2	10.2	11.1	11.1	11.1	*(25)*
Fuel oil	€/MWh_{th}	52.5	35.2	39.8	44.4	43.9	43.5	43.5	43.5	*(8)*
Fossil gas	€/MWh_{th}	21.8	22.2	30.0	32.7	36.1	40.2	40.2	40.2	*(25)*
Uranium	€/MWh_{th}	2.6	2.6	2.6	2.6	2.6	2.6	2.6	2.6	*(26)*
GHG emissions	€/t_{CO2eq}	9	28	52	61	68	75	100	150	*(25)*

GHG emissions by fuel type		
t_{CO2eq}/MWh_{th}		
Coal (27)	*Fuel oil (27)*	*Fossil gas (28)*
0.34	0.25	0.21

Supplementary Table 8. Efficiency assumptions for HVDC and HVAC transmission[29].

Component	*Power losses*
HVDC line	1.6 % / 1000 km
HVDC converter pair	1.4%
HVAC line	9.4 % / 1000 km

Supplementary Table 9. Global - Installed capacities of power and storage technologies during the energy transition from 2015 to 2050.

**Installed capacity**	**Units**	**2015**	**2020**	**2025**	**2030**	**2035**	**2040**	**2045**	**2050**
PV optimally tilted	[GW]	131	131	131	142	243	437	854	1804
PV single-axis	[GW]	0	672	2137	3982	6069	7860	9935	12117
PV prosumer	[GW]	100	365	1245	2856	4127	5508	6675	8038
Wind onshore	[GW]	364	739	2454	3286	3442	3395	3329	3154
Wind offshore	[GW]	8	8	8	7	5	0	0	0
Hydro Run-of-River	[GW]	328	328	332	332	332	332	332	332
Hydro reservoir (dam)	[GW]	700	809	892	911	928	935	939	950
Biomass Solid	[GW]	63	68	121	139	141	135	132	150
Waste-to-energy	[GW]	16	35	35	35	35	35	36	37
Biogas	[GW]	13	48	58	80	88	111	118	142
Geothermal	[GW]	13	39	64	67	69	69	67	67
CSP solar field	[GW]	5	5	6	6	6	5	8	43
CCGT	[GW]	892	968	974	960	915	818	625	541
OCGT	[GW]	897	860	1789	1773	1720	1645	1511	1536
Steam Turbine	[GW]	14	2	10	24	80	81	57	55
Internal Combustion Engine	[GW]	368	237	88	64	24	0	0	0
Coal PP	[GW]	1896	1665	1435	1293	1181	1083	955	754
Nuclear PP	[GW]	368	331	277	182	96	69	49	26
Power-to-Gas (PtG)	[GW_{el}]	0	3	2	9	112	258	521	661
Power-to-Heat (PtH)	[GW_{th}]	0	35	118	151	341	383	441	946
Battery Storage large-scale	[GWh]	2	15	259	4514	10382	15989	23304	32313
Battery Storage prosumer	[GWh]	0	203	1708	5420	8058	10767	13044	15545
Gas Storage	[GWh]	0	11931	39931	102062	220581	432784	765826	1001898
Pumped Hydro Storage (PHS)	[GWh]	135	144	225	264	265	265	265	265
A-CAES	[GWh]	0	107	143	199	202	224	328	998
Thermal Energy Storage (TES)	[GWh]	1149	1213	1268	1492	3425	3548	2429	2747

Supplementary Table 10. Global - Generation of electricity and storage output during the energy transition from 2015 to 2050.

Generation	Units	2015	2020	2025	2030	2035	2040	2045	2050
PV optimally tilted	[TWh]	187	187	188	205	372	673	1298	2647
PV single-axis	[TWh]	0	1373	4278	7848	11862	15255	19230	23570
PV prosumer	[TWh]	131	513	1788	4173	6051	8111	9867	11912
Wind onshore	[TWh]	970	2341	8059	10485	11052	10993	10816	10156
Wind offshore	[TWh]	32	32	32	29	20	0	0	0
Hydro Run-of-River	[TWh]	1166	1167	1184	1185	1185	1184	1185	1185
Hydro reservoir (dam)	[TWh]	2513	2966	3243	3305	3369	3400	3419	3453
Biomass Solid	[TWh]	172	405	821	847	819	740	673	638
Waste-to-energy	[TWh]	73	260	279	284	287	290	300	298
Biogas	[TWh]	76	294	286	268	268	276	321	345
Geothermal	[TWh]	34	259	466	478	476	475	471	470
CCGT	[TWh]	3344	4425	2302	1301	881	836	785	720
OCGT	[TWh]	2652	193	112	48	53	72	185	284
Steam Turbine	[TWh]	4	6	34	70	223	231	171	180
Internal Combustion Engine	[TWh]	867	3	0	0	0	0	0	0
Coal PP	[TWh]	9358	8830	3658	1422	556	256	83	0
Nuclear PP	[TWh]	2451	2466	2062	1355	718	513	364	194
Power-to-Gas (PtG)	[TWh_{el}]	0	3	1	18	264	602	1144	1474
Power-to-Heat (PtH)	[TWh_{th}]	0	6	83	183	591	604	436	410
Battery Storage large-scale	[TWh]	2	8	90	1472	3289	5014	7257	10098
Battery Storage prosumer	[TWh]	0	56	481	1549	2282	3026	3636	4294
Gas Storage	[TWh]	0	1	0	7	95	216	387	481
Pumped Hydro Storage (PHS)	[TWh]	27	31	55	63	57	58	54	53
A-CAES	[TWh]	0	2	2	4	4	5	8	21
Thermal Energy Storage (TES)	[TWh]	9	13	80	168	524	532	390	411

Supplementary Table 11. Europe - Installed capacities of power and storage technologies during the energy transition from 2015 to 2050.

Installed capacity	Units	2015	2020	2025	2030	2035	2040	2045	2050
PV optimally tilted	[GW]	49	49	49	52	57	132	197	217
PV single-axis	[GW]	0	90	150	229	347	409	449	472
PV prosumer	[GW]	54	135	362	590	781	974	1124	1268
Wind onshore	[GW]	127	191	355	480	537	546	552	560
Wind offshore	[GW]	8	8	8	7	5	0	0	0
Hydro Run-of-River	[GW]	67	67	67	67	67	67	67	67
Hydro reservoir (dam)	[GW]	126	129	143	147	156	157	157	157
Biomass Solid	[GW]	20	19	28	31	32	33	35	41
Waste-to-energy	[GW]	8	17	19	19	19	19	20	20
Biogas	[GW]	9	21	25	39	41	56	57	67
Geothermal	[GW]	2	3	5	5	6	6	6	6
CSP solar field	[GW]	2	2	2	2	2	2	0	0
CCGT	[GW]	177	172	168	173	166	137	111	95
OCGT	[GW]	96	94	130	145	141	133	118	130
Steam Turbine	[GW]	0	0	1	1	7	8	7	6
Internal Combustion Engine	[GW]	55	33	7	3	1	0	0	0
Coal PP	[GW]	234	166	111	72	51	36	24	20
Nuclear PP	[GW]	138	133	105	58	22	13	8	2
Power-to-Gas (PtG)	[GW_{el}]	0	0	0	0	4	20	44	45
Power-to-Heat (PtH)	[GW_{th}]	0	1	5	5	24	30	31	31
Battery Storage large-scale	[GWh]	0	0	5	156	538	1025	1434	1715
Battery Storage prosumer	[GWh]	0	111	544	933	1224	1486	1695	1854
Gas Storage	[GWh]	0	5362	24979	68267	95766	141174	196765	217330
Pumped Hydro Storage (PHS)	[GWh]	48	49	88	88	88	88	88	88
A-CAES	[GWh]	0	2	13	13	15	25	119	198
Thermal Energy Storage (TES)	[GWh]	22	23	32	33	234	290	269	268

Supplementary Table 12. Europe - Generation of electricity and storage output during the energy transition from 2015 to 2050.

Generation	Units	2015	2020	2025	2030	2035	2040	2045	2050
PV optimally tilted	[TWh]	60	60	61	64	70	178	267	300
PV single-axis	[TWh]	0	172	279	397	596	693	758	788
PV prosumer	[TWh]	65	171	450	729	962	1195	1376	1546
Wind onshore	[TWh]	324	587	1075	1425	1642	1711	1749	1773
Wind offshore	[TWh]	32	32	32	29	20	0	0	0
Hydro Run-of-River	[TWh]	192	192	192	192	192	192	192	192
Hydro reservoir (dam)	[TWh]	364	375	425	439	462	464	466	467
Biomass Solid	[TWh]	53	96	173	187	177	170	177	192
Waste-to-energy	[TWh]	47	141	151	154	156	160	166	164
Biogas	[TWh]	51	136	127	107	103	107	120	127
Geothermal	[TWh]	12	17	31	34	37	37	36	36
CCGT	[TWh]	432	479	286	292	307	292	287	279
OCGT	[TWh]	200	6	9	24	28	35	37	40
Steam Turbine	[TWh]	2	2	2	3	16	19	16	15
Internal Combustion Engine	[TWh]	63	0	0	0	0	0	0	0
Coal PP	[TWh]	1152	774	328	168	83	26	7	0
Nuclear PP	[TWh]	982	989	779	432	162	97	56	12
Power-to-Gas (PtG)	[TWh_{el}]	0	0	0	0	8	39	85	87
Power-to-Heat (PtH)	[TWh_{th}]	0	0	2	2	39	46	43	38
Battery Storage large-scale	[TWh]	0	0	2	47	149	275	379	453
Battery Storage prosumer	[TWh]	0	30	140	233	296	349	388	416
Gas Storage	[TWh]	0	0	0	0	3	14	30	30
Pumped Hydro Storage (PHS)	[TWh]	6	10	19	16	15	16	15	15
A-CAES	[TWh]	0	0	0	0	0	0	3	5
Thermal Energy Storage (TES)	[TWh]	4	4	6	6	38	43	37	33

Supplementary Table 13. Eurasia - Installed capacities of power and storage technologies during the energy transition from 2015 to 2050.

Installed capacity	Units	2015	2020	2025	2030	2035	2040	2045	2050
PV optimally tilted	[GW]	0	0	0	0	6	13	18	31
PV single-axis	[GW]	0	7	33	94	121	147	167	187
PV prosumer	[GW]	0	0	4	13	28	52	87	134
Wind onshore	[GW]	0	18	173	208	231	243	254	267
Wind offshore	[GW]	0	0	0	0	0	0	0	0
Hydro Run-of-River	[GW]	20	20	20	20	20	20	20	20
Hydro reservoir (dam)	[GW]	53	54	62	64	68	69	69	71
Biomass Solid	[GW]	0	1	2	3	3	4	4	4
Waste-to-energy	[GW]	0	1	1	1	1	1	1	1
Biogas	[GW]	0	1	1	1	2	2	2	2
Geothermal	[GW]	0	7	10	10	12	12	12	12
CSP solar field	[GW]	0	0	0	0	0	0	0	0
CCGT	[GW]	30	39	35	33	33	33	32	28
OCGT	[GW]	126	84	179	167	164	162	157	149
Steam Turbine	[GW]	0	0	0	0	0	0	0	0
Internal Combustion Engine	[GW]	9	5	1	1	0	0	0	0
Coal PP	[GW]	67	45	21	12	7	6	5	4
Nuclear PP	[GW]	24	21	17	10	5	4	4	2
Power-to-Gas (PtG)	[GW_{el}]	0	0	0	1	1	9	23	33
Power-to-Heat (PtH)	[GW_{th}]	0	0	0	0	0	1	1	1
Battery Storage large-scale	[GWh]	2	2	2	27	93	172	224	307
Battery Storage prosumer	[GWh]	0	0	0	0	4	19	72	156
Gas Storage	[GWh]	0	203	1373	4694	6516	16283	32873	49338
Pumped Hydro Storage (PHS)	[GWh]	4	4	4	4	4	4	4	4
A-CAES	[GWh]	0	0	1	1	1	1	2	64
Thermal Energy Storage (TES)	[GWh]	0	0	0	1	4	4	4	6

Supplementary Table 14. Eurasia - Generation of electricity and storage output during the energy transition from 2015 to 2050.

**Generation**	**Units**	**2015**	**2020**	**2025**	**2030**	**2035**	**2040**	**2045**	**2050**
PV optimally tilted	[TWh]	0	0	0	0	8	19	26	43
PV single-axis	[TWh]	0	12	54	147	186	226	258	288
PV prosumer	[TWh]	0	0	4	15	33	62	103	156
Wind onshore	[TWh]	0	57	537	649	723	761	797	838
Wind offshore	[TWh]	0	0	0	0	0	0	0	0
Hydro Run-of-River	[TWh]	70	70	70	70	70	70	70	70
Hydro reservoir (dam)	[TWh]	177	182	216	223	234	238	240	246
Biomass Solid	[TWh]	0	8	16	16	17	16	17	17
Waste-to-energy	[TWh]	0	9	10	10	10	10	10	10
Biogas	[TWh]	0	4	4	4	5	5	5	5
Geothermal	[TWh]	0	59	79	80	81	81	80	81
CCGT	[TWh]	89	259	67	41	30	25	27	24
OCGT	[TWh]	368	13	3	5	5	6	7	11
Steam Turbine	[TWh]	0	0	0	0	0	0	0	0
Internal Combustion Engine	[TWh]	11	0	0	0	0	0	0	0
Coal PP	[TWh]	215	320	51	12	6	3	1	0
Nuclear PP	[TWh]	178	160	129	77	35	28	28	14
Power-to-Gas (PtG)	[TWh_{el}]	0	0	0	3	4	21	52	74
Power-to-Heat (PtH)	[TWh_{th}]	0	0	0	0	0	0	0	1
Battery Storage large-scale	[TWh]	2	0	1	8	27	52	68	90
Battery Storage prosumer	[TWh]	0	0	0	0	1	5	16	35
Gas Storage	[TWh]	0	0	0	1	1	7	17	24
Pumped Hydro Storage (PHS)	[TWh]	0	1	1	1	1	1	1	1
A-CAES	[TWh]	0	0	0	0	0	0	0	1
Thermal Energy Storage (TES)	[TWh]	0	0	0	0	0	0	0	0

Supplementary Table 15. MENA - Installed capacities of power and storage technologies during the energy transition from 2015 to 2050.

Installed capacity	Units	2015	2020	2025	2030	2035	2040	2045	2050
PV optimally tilted	[GW]	1	1	1	7	76	135	182	268
PV single-axis	[GW]	0	69	218	370	556	634	670	753
PV prosumer	[GW]	0	4	18	52	97	176	294	386
Wind onshore	[GW]	2	3	111	240	240	239	239	237
Wind offshore	[GW]	0	0	0	0	0	0	0	0
Hydro Run-of-River	[GW]	5	5	5	5	5	5	5	5
Hydro reservoir (dam)	[GW]	14	15	16	16	16	16	16	16
Biomass Solid	[GW]	0	3	5	7	8	9	9	9
Waste-to-energy	[GW]	0	1	1	1	1	1	1	1
Biogas	[GW]	0	1	1	2	2	2	2	3
Geothermal	[GW]	0	0	5	5	5	5	5	6
CSP solar field	[GW]	0	0	1	1	1	1	7	40
CCGT	[GW]	97	163	171	168	164	159	146	110
OCGT	[GW]	141	132	304	300	292	279	259	227
Steam Turbine	[GW]	0	0	0	2	10	9	8	12
Internal Combustion Engine	[GW]	78	54	27	22	9	0	0	0
Coal PP	[GW]	7	7	7	5	5	3	2	1
Nuclear PP	[GW]	1	1	1	1	1	1	1	1
Power-to-Gas (PtG)	[GW_{el}]	0	0	0	0	23	48	71	83
Power-to-Heat (PtH)	[GW_{th}]	0	0	3	8	35	40	96	357
Battery Storage large-scale	[GWh]	0	0	19	548	1380	1863	2141	2641
Battery Storage prosumer	[GWh]	0	3	27	110	207	387	726	952
Gas Storage	[GWh]	0	15	189	977	9619	42949	70503	92575
Pumped Hydro Storage (PHS)	[GWh]	1	1	2	2	2	2	2	2
A-CAES	[GWh]	0	0	12	12	12	20	27	53
Thermal Energy Storage (TES)	[GWh]	2	2	12	57	341	342	361	676

Supplementary Table 16. MENA - Generation of electricity and storage output during the energy transition from 2015 to 2050.

Generation	Units	2015	2020	2025	2030	2035	2040	2045	2050
PV optimally tilted	[TWh]	2	2	2	12	131	232	314	465
PV single-axis	[TWh]	0	161	512	860	1301	1486	1574	1770
PV prosumer	[TWh]	1	6	31	91	173	314	525	689
Wind onshore	[TWh]	4	7	315	662	661	660	658	654
Wind offshore	[TWh]	0	0	0	0	0	0	0	0
Hydro Run-of-River	[TWh]	15	15	15	15	15	15	15	15
Hydro reservoir (dam)	[TWh]	38	45	47	48	48	48	48	48
Biomass Solid	[TWh]	0	23	41	41	42	42	40	30
Waste-to-energy	[TWh]	0	5	5	5	5	5	5	5
Biogas	[TWh]	0	6	6	6	6	6	6	6
Geothermal	[TWh]	0	0	39	39	39	39	39	39
CCGT	[TWh]	419	1163	696	316	114	87	90	77
OCGT	[TWh]	519	51	13	0	0	0	1	6
Steam Turbine	[TWh]	0	0	1	5	28	25	25	51
Internal Combustion Engine	[TWh]	319	0	0	0	0	0	0	0
Coal PP	[TWh]	46	60	45	28	14	7	2	0
Nuclear PP	[TWh]	4	7	7	7	7	7	7	7
Power-to-Gas (PtG)	[TWh_{el}]	0	0	0	1	53	118	163	198
Power-to-Heat (PtH)	[TWh_{th}]	0	0	0	11	72	64	53	55
Battery Storage large-scale	[TWh]	0	0	6	173	439	606	687	866
Battery Storage prosumer	[TWh]	0	1	8	35	64	120	227	296
Gas Storage	[TWh]	0	0	0	0	20	43	61	70
Pumped Hydro Storage (PHS)	[TWh]	0	0	1	0	0	0	0	0
A-CAES	[TWh]	0	0	0	0	0	0	1	1
Thermal Energy Storage (TES)	[TWh]	0	0	2	11	64	57	56	116

Supplementary Table 17. Sub-Saharan Africa - Installed capacities of power and storage technologies during the energy transition from 2015 to 2050.

Installed capacity	Units	2015	2020	2025	2030	2035	2040	2045	2050
PV optimally tilted	[GW]	1	1	1	1	1	1	1	0
PV single-axis	[GW]	0	30	78	179	302	422	643	926
PV prosumer	[GW]	0	2	15	48	98	178	263	373
Wind onshore	[GW]	1	5	50	71	73	75	77	78
Wind offshore	[GW]	0	0	0	0	0	0	0	0
Hydro Run-of-River	[GW]	5	5	6	6	6	6	6	6
Hydro reservoir (dam)	[GW]	14	20	24	28	30	33	34	34
Biomass Solid	[GW]	0	0	2	2	2	2	2	5
Waste-to-energy	[GW]	0	0	0	0	0	0	0	0
Biogas	[GW]	0	1	1	2	3	3	3	3
Geothermal	[GW]	0	0	2	2	2	2	2	2
CSP solar field	[GW]	0	0	0	0	0	0	0	0
CCGT	[GW]	8	16	21	21	21	21	21	19
OCGT	[GW]	16	23	76	75	74	76	80	85
Steam Turbine	[GW]	0	0	0	1	6	7	6	4
Internal Combustion Engine	[GW]	10	9	8	7	2	0	0	0
Coal PP	[GW]	43	39	31	26	14	10	5	5
Nuclear PP	[GW]	2	2	2	0	0	0	0	0
Power-to-Gas (PtG)	[GW_{el}]	0	0	0	0	3	9	43	51
Power-to-Heat (PtH)	[GW_{th}]	0	0	0	2	20	22	23	144
Battery Storage large-scale	[GWh]	0	0	31	330	679	978	1552	2405
Battery Storage prosumer	[GWh]	0	1	25	96	206	416	603	833
Gas Storage	[GWh]	0	32	201	466	3963	6742	39064	54013
Pumped Hydro Storage (PHS)	[GWh]	2	2	3	3	3	3	3	3
A-CAES	[GWh]	0	0	0	0	0	1	2	27
Thermal Energy Storage (TES)	[GWh]	0	0	0	22	200	224	224	236

Supplementary Table 18. Sub-Saharan Africa - Generation of electricity and storage output during the energy transition from 2015 to 2050.

Generation	Units	2015	2020	2025	2030	2035	2040	2045	2050
PV optimally tilted	[TWh]	3	3	3	3	3	2	2	0
PV single-axis	[TWh]	0	70	176	406	680	944	1429	2061
PV prosumer	[TWh]	0	3	26	83	167	305	448	632
Wind onshore	[TWh]	2	17	155	222	227	235	242	243
Wind offshore	[TWh]	0	0	0	0	0	0	0	0
Hydro Run-of-River	[TWh]	25	26	28	28	29	29	29	29
Hydro reservoir (dam)	[TWh]	65	96	113	129	143	157	161	167
Biomass Solid	[TWh]	0	2	14	13	12	10	9	12
Waste-to-energy	[TWh]	0	4	4	4	4	4	4	4
Biogas	[TWh]	0	3	3	3	3	4	4	4
Geothermal	[TWh]	0	0	13	15	15	15	15	15
CCGT	[TWh]	52	103	65	46	13	17	20	19
OCGT	[TWh]	48	13	7	1	3	7	16	21
Steam Turbine	[TWh]	0	0	0	2	16	20	20	15
Internal Combustion Engine	[TWh]	30	0	0	0	0	0	0	0
Coal PP	[TWh]	254	250	136	53	28	13	0	0
Nuclear PP	[TWh]	11	14	14	0	0	0	0	0
Power-to-Gas (PtG)	[TWh_{el}]	0	0	0	0	6	23	91	119
Power-to-Heat (PtH)	[TWh_{th}]	0	0	0	5	44	53	52	53
Battery Storage large-scale	[TWh]	0	0	11	103	214	313	501	782
Battery Storage prosumer	[TWh]	0	0	8	30	64	129	187	257
Gas Storage	[TWh]	0	0	0	0	2	7	29	37
Pumped Hydro Storage (PHS)	[TWh]	0	1	1	1	1	1	1	1
A-CAES	[TWh]	0	0	0	0	0	0	0	1
Thermal Energy Storage (TES)	[TWh]	0	0	0	5	38	46	45	34

Supplementary Table 19. SAARC - Installed capacities of power and storage technologies during the energy transition from 2015 to 2050.

Installed capacity	Units	2015	2020	2025	2030	2035	2040	2045	2050
PV optimally tilted	[GW]	6	6	6	9	20	40	71	88
PV single-axis	[GW]	0	87	369	708	1078	1431	1915	2505
PV prosumer	[GW]	0	12	88	273	439	648	856	1137
Wind onshore	[GW]	24	25	61	172	169	166	176	200
Wind offshore	[GW]	0	0	0	0	0	0	0	0
Hydro Run-of-River	[GW]	21	21	21	21	21	21	21	21
Hydro reservoir (dam)	[GW]	33	47	48	49	49	49	49	50
Biomass Solid	[GW]	6	8	17	24	27	28	27	27
Waste-to-energy	[GW]	0	2	3	3	3	3	3	3
Biogas	[GW]	0	10	11	11	12	12	11	12
Geothermal	[GW]	0	0	5	5	5	5	5	5
CSP solar field	[GW]	0	0	0	0	0	0	0	0
CCGT	[GW]	46	53	56	53	51	46	43	37
OCGT	[GW]	16	57	118	117	114	113	112	111
Steam Turbine	[GW]	0	0	0	1	13	14	13	12
Internal Combustion Engine	[GW]	15	12	5	4	1	0	0	0
Coal PP	[GW]	163	160	152	142	127	115	106	96
Nuclear PP	[GW]	6	7	6	6	6	5	4	3
Power-to-Gas (PtG)	[GW_{el}]	0	0	0	0	1	2	44	69
Power-to-Heat (PtH)	[GW_{th}]	0	0	0	2	46	50	50	50
Battery Storage large-scale	[GWh]	0	0	87	1321	2465	3698	5175	6964
Battery Storage prosumer	[GWh]	0	0	138	609	962	1375	1753	2227
Gas Storage	[GWh]	0	406	530	2224	11079	19723	60504	90806
Pumped Hydro Storage (PHS)	[GWh]	4	11	44	44	44	44	44	44
A-CAES	[GWh]	0	0	0	0	1	1	1	1
Thermal Energy Storage (TES)	[GWh]	3	4	4	20	464	500	497	497

Supplementary Table 20. SAARC - Generation of electricity and storage output during the energy transition from 2015 to 2050.

Generation	Units	2015	2020	2025	2030	2035	2040	2045	2050
PV optimally tilted	[TWh]	10	10	10	15	33	68	120	150
PV single-axis	[TWh]	0	180	766	1452	2191	2895	3857	5033
PV prosumer	[TWh]	0	19	141	438	704	1040	1373	1822
Wind onshore	[TWh]	40	44	130	383	379	375	403	456
Wind offshore	[TWh]	0	0	0	0	0	0	0	0
Hydro Run-of-River	[TWh]	81	81	81	81	81	81	81	81
Hydro reservoir (dam)	[TWh]	121	177	179	180	180	180	181	182
Biomass Solid	[TWh]	14	70	140	172	168	154	138	123
Waste-to-energy	[TWh]	1	19	21	22	22	22	22	22
Biogas	[TWh]	1	32	34	34	35	35	35	35
Geothermal	[TWh]	0	0	40	40	40	40	40	40
CCGT	[TWh]	199	208	142	62	34	32	43	40
OCGT	[TWh]	52	38	52	1	4	5	16	32
Steam Turbine	[TWh]	0	0	0	2	37	37	36	34
Internal Combustion Engine	[TWh]	74	0	0	0	0	0	0	0
Coal PP	[TWh]	1093	1156	761	252	76	29	3	0
Nuclear PP	[TWh]	34	49	48	45	43	37	29	26
Power-to-Gas (PtG)	[TWh_{el}]	0	0	0	0	1	6	93	156
Power-to-Heat (PtH)	[TWh_{th}]	0	0	0	4	100	97	95	89
Battery Storage large-scale	[TWh]	0	0	33	430	793	1158	1617	2162
Battery Storage prosumer	[TWh]	0	0	41	181	284	406	516	655
Gas Storage	[TWh]	0	0	0	0	1	2	32	50
Pumped Hydro Storage (PHS)	[TWh]	1	3	13	12	11	10	10	10
A-CAES	[TWh]	0	0	0	0	0	0	0	0
Thermal Energy Storage (TES)	[TWh]	1	1	1	4	87	85	82	77

Supplementary Table 21. Northeast Asia - Installed capacities of power and storage technologies during the energy transition from 2015 to 2050.

**Installed capacity**	**Units**	**2015**	**2020**	**2025**	**2030**	**2035**	**2040**	**2045**	**2050**
PV optimally tilted	[GW]	52	52	52	52	63	89	160	775
PV single-axis	[GW]	0	143	592	1189	1919	2650	3546	4271
PV prosumer	[GW]	28	114	316	880	1304	1716	2000	2371
Wind onshore	[GW]	118	322	1018	1209	1211	1169	1102	921
Wind offshore	[GW]	0	0	0	0	0	0	0	0
Hydro Run-of-River	[GW]	88	88	88	89	89	89	89	89
Hydro reservoir (dam)	[GW]	205	267	302	302	302	302	302	305
Biomass Solid	[GW]	8	9	28	33	34	32	30	33
Waste-to-energy	[GW]	3	5	5	5	4	4	4	4
Biogas	[GW]	1	3	5	7	8	8	9	16
Geothermal	[GW]	1	1	3	4	4	4	4	5
CSP solar field	[GW]	0	0	0	0	0	0	0	2
CCGT	[GW]	111	110	106	104	97	87	81	84
OCGT	[GW]	140	135	252	250	243	230	226	299
Steam Turbine	[GW]	13	0	7	15	22	21	4	3
Internal Combustion Engine	[GW]	90	53	10	6	3	0	0	0
Coal PP	[GW]	942	930	918	902	870	824	739	568
Nuclear PP	[GW]	80	79	74	63	51	39	30	18
Power-to-Gas (PtG)	[GW_{el}]	0	0	0	4	8	31	90	148
Power-to-Heat (PtH)	[GW_{th}]	0	2	73	87	108	109	109	109
Battery Storage large-scale	[GWh]	0	1	7	897	2581	4374	7179	10747
Battery Storage prosumer	[GWh]	0	60	245	1571	2591	3535	4158	4960
Gas Storage	[GWh]	0	3076	3738	8194	23693	42786	108734	185428
Pumped Hydro Storage (PHS)	[GWh]	56	56	61	98	98	98	98	98
A-CAES	[GWh]	0	6	13	66	67	69	70	329
Thermal Energy Storage (TES)	[GWh]	1104	1107	1111	1144	1358	1360	262	260

Supplementary Table 22. Northeast Asia - Generation of electricity and storage output during the energy transition from 2015 to 2050.

**Generation**	**Units**	**2015**	**2020**	**2025**	**2030**	**2035**	**2040**	**2045**	**2050**
PV optimally tilted	[TWh]	80	80	80	80	96	134	239	1070
PV single-axis	[TWh]	0	279	1077	2163	3450	4737	6331	7688
PV prosumer	[TWh]	38	161	452	1290	1912	2520	2940	3486
Wind onshore	[TWh]	341	1026	3368	4000	4013	3899	3700	3087
Wind offshore	[TWh]	0	0	0	0	0	0	0	0
Hydro Run-of-River	[TWh]	309	309	309	310	310	310	310	310
Hydro reservoir (dam)	[TWh]	724	970	1073	1075	1075	1075	1075	1081
Biomass Solid	[TWh]	37	70	220	220	214	195	173	158
Waste-to-energy	[TWh]	11	29	31	31	31	31	34	33
Biogas	[TWh]	4	26	26	27	29	29	29	30
Geothermal	[TWh]	4	8	22	29	28	28	29	29
CCGT	[TWh]	566	298	114	82	58	77	94	89
OCGT	[TWh]	485	25	13	11	6	2	24	58
Steam Turbine	[TWh]	0	0	25	43	59	60	12	9
Internal Combustion Engine	[TWh]	162	0	0	0	0	0	0	0
Coal PP	[TWh]	4322	3828	1482	552	226	120	45	0
Nuclear PP	[TWh]	287	587	552	467	381	288	222	136
Power-to-Gas (PtG)	[TWh_{el}]	0	0	0	6	16	66	193	306
Power-to-Heat (PtH)	[TWh_{th}]	0	0	70	121	161	163	32	21
Battery Storage large-scale	[TWh]	0	0	3	304	823	1387	2251	3354
Battery Storage prosumer	[TWh]	0	17	70	457	746	1004	1167	1366
Gas Storage	[TWh]	0	0	0	2	6	24	67	100
Pumped Hydro Storage (PHS)	[TWh]	14	10	14	26	23	24	21	19
A-CAES	[TWh]	0	0	0	1	1	2	2	6
Thermal Energy Storage (TES)	[TWh]	0	0	59	103	139	141	28	20

Supplementary Table 23. Southeast Asia - Installed capacities of power and storage technologies during the energy transition from 2015 to 2050.

Installed capacity	Units	2015	2020	2025	2030	2035	2040	2045	2050
PV optimally tilted	[GW]	2	2	2	2	2	10	171	331
PV single-axis	[GW]	0	48	240	499	823	1025	1186	1403
PV prosumer	[GW]	5	21	73	221	325	443	547	685
Wind onshore	[GW]	5	12	43	71	71	69	74	80
Wind offshore	[GW]	0	0	0	0	0	0	0	0
Hydro Run-of-River	[GW]	18	18	21	21	21	21	21	21
Hydro reservoir (dam)	[GW]	35	44	45	46	46	46	46	48
Biomass Solid	[GW]	5	6	14	16	15	14	16	24
Waste-to-energy	[GW]	0	2	3	3	2	2	2	3
Biogas	[GW]	1	3	5	6	8	9	10	11
Geothermal	[GW]	4	8	14	14	14	14	13	14
CSP solar field	[GW]	0	0	0	0	0	0	0	0
CCGT	[GW]	53	55	73	73	69	57	46	47
OCGT	[GW]	55	59	99	98	87	77	72	74
Steam Turbine	[GW]	0	0	0	1	17	17	14	14
Internal Combustion Engine	[GW]	17	12	7	6	2	0	0	0
Coal PP	[GW]	80	76	71	60	54	49	40	32
Nuclear PP	[GW]	0	0	0	0	0	0	0	0
Power-to-Gas (PtG)	[GW_{el}]	0	0	0	0	0	7	38	59
Power-to-Heat (PtH)	[GW_{th}]	0	0	0	5	58	58	58	180
Battery Storage large-scale	[GWh]	0	0	93	757	1651	2208	3001	3986
Battery Storage prosumer	[GWh]	0	12	97	477	686	902	1084	1303
Gas Storage	[GWh]	0	302	836	4004	12729	26895	56822	80533
Pumped Hydro Storage (PHS)	[GWh]	4	4	7	8	8	8	8	8
A-CAES	[GWh]	0	0	0	0	0	0	0	215
Thermal Energy Storage (TES)	[GWh]	0	0	0	52	583	584	584	615

Supplementary Table 24. Southeast Asia - Generation of electricity and storage output during the energy transition from 2015 to 2050.

**Generation**	**Units**	**2015**	**2020**	**2025**	**2030**	**2035**	**2040**	**2045**	**2050**
PV optimally tilted	[TWh]	4	4	4	4	4	15	251	485
PV single-axis	[TWh]	0	93	456	948	1551	1932	2244	2667
PV prosumer	[TWh]	8	34	117	341	497	677	833	1041
Wind onshore	[TWh]	14	40	139	197	196	190	196	198
Wind offshore	[TWh]	0	0	0	0	0	0	0	0
Hydro Run-of-River	[TWh]	66	66	81	81	81	81	81	81
Hydro reservoir (dam)	[TWh]	128	168	173	173	173	173	173	179
Biomass Solid	[TWh]	7	32	95	97	94	86	75	62
Waste-to-energy	[TWh]	1	19	20	20	21	21	21	21
Biogas	[TWh]	5	22	22	23	24	24	28	29
Geothermal	[TWh]	2	34	83	83	83	83	83	83
CCGT	[TWh]	259	364	261	136	43	43	50	49
OCGT	[TWh]	218	24	12	3	3	7	13	27
Steam Turbine	[TWh]	0	0	0	4	50	53	47	44
Internal Combustion Engine	[TWh]	45	0	0	0	0	0	0	0
Coal PP	[TWh]	483	578	333	179	38	33	22	0
Nuclear PP	[TWh]	0	0	0	0	0	0	0	0
Power-to-Gas (PtG)	[TWh_{el}]	0	0	1	0	1	15	75	131
Power-to-Heat (PtH)	[TWh_{th}]	0	0	0	11	134	138	124	118
Battery Storage large-scale	[TWh]	0	0	28	251	537	711	962	1291
Battery Storage prosumer	[TWh]	0	4	31	145	209	275	330	397
Gas Storage	[TWh]	0	0	0	0	0	5	26	44
Pumped Hydro Storage (PHS)	[TWh]	1	1	2	2	2	2	2	2
A-CAES	[TWh]	0	0	0	0	0	0	0	5
Thermal Energy Storage (TES)	[TWh]	0	0	0	9	117	120	108	100

Supplementary Table 25. North America - Installed capacities of power and storage technologies during the energy transition from 2015 to 2050.

Installed capacity	Units	2015	2020	2025	2030	2035	2040	2045	2050
PV optimally tilted	[GW]	16	16	16	16	16	15	52	95
PV single-axis	[GW]	0	120	327	546	725	887	1011	1150
PV prosumer	[GW]	12	66	301	634	847	1039	1172	1302
Wind onshore	[GW]	78	122	599	789	862	839	810	766
Wind offshore	[GW]	0	0	0	0	0	0	0	0
Hydro Run-of-River	[GW]	64	64	64	64	64	64	64	64
Hydro reservoir (dam)	[GW]	110	111	127	134	135	135	136	138
Biomass Solid	[GW]	10	9	10	9	9	8	6	6
Waste-to-energy	[GW]	5	5	4	4	4	4	4	4
Biogas	[GW]	3	5	5	7	8	12	14	17
Geothermal	[GW]	5	19	21	20	19	19	18	18
CSP solar field	[GW]	2	2	2	2	2	2	1	0
CCGT	[GW]	336	326	310	301	282	252	127	107
OCGT	[GW]	271	222	583	575	559	533	450	433
Steam Turbine	[GW]	0	1	1	3	5	5	4	3
Internal Combustion Engine	[GW]	66	36	6	3	0	0	0	0
Coal PP	[GW]	348	231	116	66	46	33	29	23
Nuclear PP	[GW]	114	86	69	43	11	6	2	0
Power-to-Gas (PtG)	[GW_{el}]	0	3	1	4	73	132	168	171
Power-to-Heat (PtH)	[GW_{th}]	0	32	32	37	44	44	44	45
Battery Storage large-scale	[GWh]	0	12	12	379	852	1403	2079	2723
Battery Storage prosumer	[GWh]	0	6	489	1311	1741	2072	2285	2494
Gas Storage	[GWh]	0	1940	6091	9899	53263	131255	194129	222194
Pumped Hydro Storage (PHS)	[GWh]	16	17	17	17	17	17	17	17
A-CAES	[GWh]	0	97	98	99	99	100	100	102
Thermal Energy Storage (TES)	[GWh]	18	77	78	133	199	201	183	146

Supplementary Table 26. North America - Generation of electricity and storage output during the energy transition from 2015 to 2050.

Generation	Units	2015	2020	2025	2030	2035	2040	2045	2050
PV optimally tilted	[TWh]	24	24	24	24	24	22	75	136
PV single-axis	[TWh]	0	250	693	1131	1500	1819	2068	2345
PV prosumer	[TWh]	18	100	458	954	1270	1554	1748	1933
Wind onshore	[TWh]	222	384	2148	2748	3005	2948	2862	2702
Wind offshore	[TWh]	0	0	0	0	0	0	0	0
Hydro Run-of-River	[TWh]	247	247	247	247	247	247	247	247
Hydro reservoir (dam)	[TWh]	423	427	482	502	503	504	509	517
Biomass Solid	[TWh]	14	42	58	47	45	38	29	28
Waste-to-energy	[TWh]	14	27	28	30	31	31	32	32
Biogas	[TWh]	14	27	24	24	25	26	33	39
Geothermal	[TWh]	16	140	157	157	152	151	149	148
CCGT	[TWh]	1130	1399	577	262	217	205	140	114
OCGT	[TWh]	637	20	0	1	1	1	66	83
Steam Turbine	[TWh]	1	3	3	10	14	14	11	9
Internal Combustion Engine	[TWh]	70	2	0	0	0	0	0	0
Coal PP	[TWh]	1718	1840	517	179	86	25	3	0
Nuclear PP	[TWh]	934	642	514	317	81	47	12	0
Power-to-Gas (PtG)	[TWh_{el}]	0	3	0	7	171	314	390	397
Power-to-Heat (PtH)	[TWh_{th}]	0	6	5	23	33	33	29	26
Battery Storage large-scale	[TWh]	0	7	4	124	260	427	623	834
Battery Storage prosumer	[TWh]	0	2	140	372	485	567	608	647
Gas Storage	[TWh]	0	1	0	3	62	114	124	124
Pumped Hydro Storage (PHS)	[TWh]	5	5	4	5	4	4	4	5
A-CAES	[TWh]	0	2	1	2	2	2	2	2
Thermal Energy Storage (TES)	[TWh]	4	7	8	24	33	32	26	22

Supplementary Table 27. South America - Installed capacities of power and storage technologies during the energy transition from 2015 to 2050.

Installed capacity	Units	2015	2020	2025	2030	2035	2040	2045	2050
PV optimally tilted	[GW]	2	2	2	2	2	2	2	0
PV single-axis	[GW]	0	78	129	169	200	256	348	452
PV prosumer	[GW]	0	11	68	145	209	280	330	383
Wind onshore	[GW]	8	41	44	45	47	49	46	44
Wind offshore	[GW]	0	0	0	0	0	0	0	0
Hydro Run-of-River	[GW]	39	39	39	39	39	39	39	39
Hydro reservoir (dam)	[GW]	112	121	125	125	127	129	130	130
Biomass Solid	[GW]	13	13	14	14	10	5	2	2
Waste-to-energy	[GW]	0	1	1	1	1	1	1	1
Biogas	[GW]	0	5	5	5	6	7	11	12
Geothermal	[GW]	1	1	1	0	0	0	0	0
CSP solar field	[GW]	0	0	0	0	0	0	0	0
CCGT	[GW]	32	34	34	34	32	25	19	13
OCGT	[GW]	35	52	49	47	45	42	36	29
Steam Turbine	[GW]	0	0	0	1	1	1	1	1
Internal Combustion Engine	[GW]	27	22	16	14	5	0	0	0
Coal PP	[GW]	10	10	8	8	7	7	5	5
Nuclear PP	[GW]	3	2	2	1	1	1	1	0
Power-to-Gas (PtG)	[GW_{el}]	0	0	0	0	0	1	1	3
Power-to-Heat (PtH)	[GW_{th}]	0	0	4	5	6	29	30	30
Battery Storage large-scale	[GWh]	0	0	3	100	144	266	520	826
Battery Storage prosumer	[GWh]	0	11	142	314	439	575	669	765
Gas Storage	[GWh]	0	595	1994	3338	3953	4977	6433	9681
Pumped Hydro Storage (PHS)	[GWh]	0	0	0	0	0	0	0	0
A-CAES	[GWh]	0	1	7	7	7	8	8	9
Thermal Energy Storage (TES)	[GWh]	0	1	31	31	43	43	44	45

Supplementary Table 28. South America - Generation of electricity and storage output during the energy transition from 2015 to 2050.

Generation	Units	2015	2020	2025	2030	2035	2040	2045	2050
PV optimally tilted	[TWh]	4	4	4	4	4	4	4	0
PV single-axis	[TWh]	0	157	265	344	406	521	711	931
PV prosumer	[TWh]	0	18	109	232	332	445	523	606
Wind onshore	[TWh]	23	180	193	199	206	215	210	204
Wind offshore	[TWh]	0	0	0	0	0	0	0	0
Hydro Run-of-River	[TWh]	162	162	161	162	162	160	161	162
Hydro reservoir (dam)	[TWh]	471	526	536	536	552	561	566	566
Biomass Solid	[TWh]	48	61	63	54	51	30	16	15
Waste-to-energy	[TWh]	0	7	7	7	7	6	7	7
Biogas	[TWh]	1	38	39	39	39	40	61	70
Geothermal	[TWh]	0	0	0	0	0	0	0	0
CCGT	[TWh]	198	152	94	65	65	59	34	29
OCGT	[TWh]	127	2	2	3	4	8	5	6
Steam Turbine	[TWh]	0	0	2	2	3	3	3	3
Internal Combustion Engine	[TWh]	94	0	0	0	0	0	0	0
Coal PP	[TWh]	74	23	5	0	0	0	0	0
Nuclear PP	[TWh]	21	18	18	9	9	9	9	0
Power-to-Gas (PtG)	[TWh_{el}]	0	0	0	2	2	2	3	5
Power-to-Heat (PtH)	[TWh_{th}]	0	0	6	6	8	9	8	9
Battery Storage large-scale	[TWh]	0	0	1	32	46	86	168	265
Battery Storage prosumer	[TWh]	0	3	44	95	132	172	198	225
Gas Storage	[TWh]	0	0	0	1	1	1	1	1
Pumped Hydro Storage (PHS)	[TWh]	0	0	0	0	0	0	0	0
A-CAES	[TWh]	0	0	0	0	0	0	0	0
Thermal Energy Storage (TES)	[TWh]	0	0	5	5	7	7	7	8

Supplementary Table 29. GHG emissions during the energy transition from 2015 to 2050.

GHG emissions	Units	2015	2020	2025	2030	2035	2040	2045	2050
Global	[Mt_{CO2eq}]	10721	8507	3580	1399	525	234	78	0
Europe	[Mt_{CO2eq}]	1226	703	272	141	75	24	5	0
Eurasia	[Mt_{CO2eq}]	402	351	62	22	13	7	3	0
MENA	[Mt_{CO2eq}]	721	494	291	132	41	17	8	0
Sub-Saharan Africa	[Mt_{CO2eq}]	269	241	133	57	26	16	2	0
SAARC	[Mt_{CO2eq}]	1027	1000	670	213	64	26	4	0
Northeast Asia	[Mt_{CO2eq}]	4007	3129	1200	452	181	95	35	0
Southeast Asia	[Mt_{CO2eq}]	627	589	351	178	33	29	18	0
North America	[Mt_{CO2eq}]	2167	1951	587	204	92	20	3	0
South America	[Mt_{CO2eq}]	275	49	14	0	0	0	0	0

Supplementary References

1. P.W. Stackhouse, C.H. Whitlock, "Surface meteorology and solar energy (SSE) release 6.0, NASA SSE 6.0" (Earth Science Enterprise Program. National Aeronautic and Space Administration (NASA), Langley, 2008; http://eosweb.larc.nasa.gov/sse/).
2. P.W. Stackhouse, C.H. Whitlock, "Surface meteorology and solar energy (SSE) release 6.0 Methodology, NASA SSE 6.0". (Earth Science Enterprise Program, National Aeronautic and Space Administration (NASA), Langley, 2009; http://eosweb.larc.nasa.gov/sse/documents/SSE6Methodology.pdf).
3. D. Stetter, "Enhancement of the REMix energy system model: global renewable energy potentials optimized power plant siting and scenario validation", dissertation, Faculty of Energy-, Process and Bio-Engineering, University of Stuttgart (2012).
4. D. Bogdanov, Ch. Breyer, North-East Asian Super Grid for 100% renewable energy supply: Optimal mix of energy technologies for electricity, gas and heat supply options. *Energy Conversion and Management* **112**, 176–190 (2016).
5. K. Verzano, "Climate Change Impacts on Flood Related Hydrological Processes: Further Development and Application of a Global Scale Hydrological Model", dissertation, Faculty of Electrical Engineering and Computer Science, University of Kassel (2009).
6. K. Bunzel, V. Zeller, M. Buchhorn, F. Griem, D. Thrän, "Regionale und globale räumliche Verteilung von Biomassepotenzialen" (German Biomass Research Center, Leipzig, 2009).
7. B. Elbersen, I. Startisky, G. Hengeveld, M.-J. Schelhaas, H. Naeff, "Atlas of EU biomass potentials," (European Commission - Biomass Futures, Brussels, 2012).
8. International Energy Agency (IEA), "World Energy Outlook 2015" (IEA, Paris, 2015).
9. K. Sadovskaia, D. Bogdanov, S. Honkapuro, Ch. Breyer, Power Transmission and Distributions Losses – A Model Based on Available Empirical Data and Future Trends for All Countries Globally. International Journal of Electrical Power & Energy Systems **107**, 98-109 (2019).
10. A. Toktarova, L. Gruber, M. Hlusiak, D. Bogdanov, Ch. Breyer, Long-term load forecasting in high resolution for all countries globally, submitted.
11. G. Pleßmann, M. Erdmann, M. Hlusiak, Ch. Breyer, Global energy storage demand for a 100% renewable electricity supply. *Energy Procedia* **46**, 22-31 (2014).
12. European Commission, "Energy Technology Reference Indicator (ETRI) Projections for 2010-2050" (European Commission. Joint Research Centre. Institute for Energy and Transport, Petten, 2014).
13. European Technology and Innovation Platform Photovoltaics, "The True Competitiveness of Solar PV. A European Case Study" (European Technology and Innovation Platform Photovoltaics, Munich, 2017).

14. M. Bolinger, J. Seel "Utility-Scale Solar 2015: An Empirical Analysis of Project Cost, Performance, and Pricing Trends in the United States" (Lawrence Berkeley National Laboratory. Berkley; 2016; https://emp.lbl.gov/publications/utility-scale-solar-2015-empirical).

15. L. Neij, Cost development of future technologies for power generation — A study based on experience curves and complementary bottom-up assessments. *Energy Policy* **36**(6), 2200-2211 (2008).
16. J. E Haysom., O. Jafarieh, H. Anis, K. Hinzer, D.Wright, Learning curve analysis of concentrated photovoltaic systems. *Prog Photovoltaics Res Appl*. **23**(11),1678-1686 (2015).
17. C. Kutscher, M. Mehos, C. Turchi, G. Glatzmaier, T. Moss "Line-Focus Solar Power Plant Cost Reduction Plan" (National Renewable Energy Laboratory (NREL). Vol NREL/TP-55. Golden, 2010).
18. B. Sigfússon, A. Uihlein, "2015 JRC Geothermal Energy Status Report" (European Commission - Joint Research Centre, Petten, 2015).
19. Agora Energiewende, "Stromspeicher in Der Energiewende" (Agora Energiewende, Berlin, 2014; https://www.agora-energiewende.de/en/topics/-agothem/Produkt/produkt/61/Stromspeicher+in+der+Energiewende/).
20. Ch. Breyer, E. Tsupari, V. Tikka, P. Vainikka, Power-to-gas as an emerging profitable business through creating an integrated value chain. *Energy Procedia* **73,** 182-189 (2015).

21. W Urban, K Girod, H Lohmann, E. Weidner, "Technologien Und Kosten Der Biogasaufbereitung Und Einspeisung in Das Erdgasnetz . Ergebnisse Der Markterhebung 2007-2008" (Fraunhofer Instituts, Fraunhofer UMSICHT, 2008; http://publica.fraunhofer.de/dokumente/N-94887.html).

22. W. Hoffmann, Importance and evidence for cost effective electricity storage. paper presented at 29th EU PVSEC. Amsterdam, September 22-26; 2014.
23. International Renewable Energy Agency (IRENA), "Electricity Storage and Renewables: Costs and Markets to 2030" (International Renewable Energy Agency,Abu Dhabi, 2017; www.irena.org).
24. A. Gambhir, L. Drouet, D. McCollum, T. Napp, D. Bernie, A. Hawkes, O. Fricko, P. Havlik, K. Riahi, V. Bosetti, J. Lowe, Assessing the feasibility of global long-term mitigation scenarios. *Energies* **10(1)**, 89 (2017).
25. Bloomberg New Energy Finance, "New Energy Outlook 2015 - Long-Term Projections of the Global Energy Sector" (Bloomberg New Energy Finance, London, 2015).
26. International Energy Agency and Nuclear Energy Agency, "Projected Costs of Generating Electricity" (International Energy Agency, Paris, 2015).
27. CSIRO Division of Atmospheric Research, "Lifecycle emissions and energy analysis of LNG, oil and coal" (CSIRO Division of Atmospheric Research, Aspendale, Australia, December 1996)
28. Environmental Protection Agency, "Annexes to the Inventory of U.S. GHG Emissions and Sinks", (Environmental Protection Agency, Washington, DC, USA, 2013)

29. F. Zickfeld, A. Wieland, “Perspectives on a Sustainable Power System for EUMENA. Desert Power 2050” (Dii GmbH. Munich, 2012; http://www.desertenergy.org).
